# Chloroquine induces eryptosis in *P. falciparum*-infected red blood cells and the release of extracellular vesicles with a unique protein profile

**DOI:** 10.3389/fcimb.2025.1553123

**Published:** 2025-05-26

**Authors:** Claudia Carrera-Bravo, Tianchi Zhou, Trang Chu, Jing Wen Hang, Harshvardhan Modh, Chenyuan Huang, Sitong Zhang, Haining Hao, María José Cabada-García, Benoit Malleret, Matthias G. Wacker, Jiong-Wei Wang, Laurent Rénia, Kevin S. W. Tan

**Affiliations:** ^1^ Department of Microbiology and Immunology, Healthy Longevity Translational Research Programme, Yong Loo Lin School of Medicine, National University of Singapore, Singapore, Singapore; ^2^ ASTAR Infectious Diseases Labs, Agency for Science, Technology and Research, Singapore, Singapore; ^3^ Centre for Inflammation Research, The Queen’s Medical Research Institute, The University of Edinburgh, Edinburgh, United Kingdom; ^4^ Medical Research Council (MRC) Human Genetics Unit, Institute of Genetics and Cancer, The University of Edinburg, Edinburgh, United Kingdom; ^5^ Department of Microbiology and Immunology, Immunology Translational Research Programme, Yoon Loo Lin School of Medicine, National University of Singapore, Singapore, Singapore; ^6^ Department of Pharmacy and Pharmaceutical Sciences, Faculty of Science, National University of Singapore, Singapore, Singapore; ^7^ Department of Surgery, Yong Loo Lin School of Medicine, National University of Singapore, Singapore, Singapore; ^8^ Nanomedicine Translational Research Programme, Yong Loo Lin School of Medicine, National University of Singapore, Singapore, Singapore; ^9^ Laboratory of Quality and Safety Risk Assessment for Agro-Products of the Ministry of Agriculture (Jinan), Institute of Quality Standard and Testing Technology for Agro-Products, Shandong Academy of Agricultural Sciences, Jinan, China; ^10^ Tecnológico de Monterrey, Escuela de Medicina y Ciencias de la Salud, Monterrey, Mexico; ^11^ Singapore Immunology Network (SIgN), Agency for Science, Technology and Research, Singapore, Singapore; ^12^ Cardiovascular Research Institute (CVRI), National University Health System, Singapore, Singapore; ^13^ Department of Physiology, Yong Loo Lin School of Medicine, National University of Singapore, Singapore, Singapore

**Keywords:** eryptosis, *Plasmodium falciparum*, chloroquine, extracellular vesicles, proteomics, host-parasite interaction

## Abstract

**Introduction:**

Malaria is a vector-borne parasitic disease that affects millions worldwide. To achieve the objective set by the World Health Organization of reducing malaria cases by 2030, antimalarial drugs with novel modes of action are required. Previously, a novel mechanism of action of chloroquine (CQ) was reported involving features of programmed cell death in the parasite, mainly characterized by calcium efflux from digestive vacuole permeabilization. Increased intracellular calcium induces suicidal death of erythrocytes, a process known as eryptosis. This study aimed to identify the hallmarks of eryptosis due to calcium redistribution and examine the downstream cellular effects during CQ treatment in infected red blood cells (iRBCs).

**Methods:**

Synchronized *Plasmodium falciparum* 3D7 cultures at mid-late trophozoites were treated with CQ and other antimalarial compounds for 10 hours. Eryptotic markers, including phosphatidylserine (PS) exposure, cell shrinkage and membrane blebbing, were assessed by flow cytometry, scanning electron microscopy and western blot, respectively. Extracellular vesicles (EVs) were isolated from 3D7 malaria culture supernatants using differential ultracentrifugation, followed by their physical and proteomic characterization. THP-1-derived macrophages were stimulated with EVs to determine parasite-host interactions, as indicated by cytokine levels and transcriptomic analysis.

**Results:**

Increased PS exposure, cell shrinkage, and membrane blebbing were observed, delineating an eryptotic phenotype in the host RBCs. Notably, the outward budding and blebbing of the iRBC plasma membrane led to the formation of EVs, which are complex structures with unique functional properties. Proteomic characterization of EVs from CQ-treated iRBCs revealed a high enrichment of proteasome and ribosome protein clusters. This unique EV cargo did not influence the parasite growth rate but might activate IFN signaling pathways mediated by IL-6 in THP-1-derived macrophages.

**Conclusion:**

These findings provide new insights into a novel drug-induced cell death mechanism that targets the parasite and specific components of the infected host RBC.

## Introduction

Malaria is considered one of the most devastating infectious diseases worldwide with an estimated 249 million cases reported in 2022. Of this number, 93.6% belongs to the WHO African Region having the highest share of the global malaria burden (‘World Malaria Report 2023’, n.d.). Therefore, efforts toward its control and eradication through novel antimalarial drugs as well as the search for the long-awaited vaccine are urgently needed. Currently, five species of the parasite are known to cause disease in humans. Among them, *P. falciparum* is associated with the highest mortality due to its prevalence, virulence and drug resistance (‘World Malaria Report 2023’, n.d.). The primary symptoms of malaria including fever, headache and chills, result from the rupture of infected red blood cells (iRBCs). This rupture causes a systemic release of pro-inflammatory cytokines and other molecules, which contributes to malaria pathogenesis (Dunst et al., 2017). Consequently, the emergence of resistance to antimalarial compounds remains a significant challenge, particularly for compounds targeting the blood stage that leads to their discontinuity for routine treatment within malaria-endemic regions.

Chloroquine (CQ) is a drug that has been used to prevent and treat malaria since the 1940s. However, its lack of efficacy in treating *P. falciparum* infections has been linked to genetic pressure on erythrocytic-stage parasites, resulting in resistance (Coban, 2020). CQ acts on *Plasmodium* late stages (trophozoites and schizonts) by entering the digestive vacuole (DV) to prevent heme polymerization into hemozoin, hence blocking the parasite detoxification process (Slater, 1993). In 2011, our group demonstrated an alternative mechanism of action *in vitro* (Ch’ng et al., 2011) which was confirmed subsequently by *ex vivo* assays, implicating its clinical relevance (Ch’ng et al., 2014). The studies showed that CQ treatment caused permeability of the parasite DV membrane resulting in calcium leakage into the iRBC cytoplasm, involving features of programmed cell death in the parasite, including mitochondrial outer membrane permeabilization and DNA degradation. However, its downstream effects on the host RBC were unknown.

It has been reported that an increase in intracellular calcium leads to the suicidal death of RBCs, a process described as eryptosis (Repsold and Joubert, 2018). Interestingly, during *Plasmodium* invasion and development, the parasite employs a strategy to delay eryptosis, allowing its survival by sequestering calcium in different compartments, including the DV (Boulet et al., 2018; Scovino et al., 2022). Therefore, we proposed that calcium homeostasis disruption in late-stage iRBCs due to CQ treatment might trigger eryptotic hallmarks. In the current study, we show that CQ-treated iRBCs displayed higher levels of phosphatidylserine (PS) externalization and ceramide abundance on the plasma membrane, cell shrinkage, and membrane blebbing; all of which are considered classical features of eryptosis.

Following the downstream cellular events of CQ-treated iRBCs, we characterized the outward blebs from the cell surface released to the extracellular milieu also known as RBC-derived extracellular vesicles (EVs). These are spheroid structures comprising a lipid bilayer membrane carrying proteins, metabolites, lipids and nucleic acids from the parasite and the host cell (Sampaio et al., 2017). Notably, RBC-derived EVs have been divided into three groups according to their size: Exosome-like vesicles (30–100 nm), microvesicles (100 – 1–000 nm) and eryptotic bodies (500 – 5–000 nm). In malaria, EVs are involved in cell-cell communication during the life cycle, immunomodulation, as stage-dependent biomarkers, among others (Carrera-Bravo et al., 2021). Studies on the roles of EVs in malaria pathogenicity are slowly emerging (Regev-Rudzki et al., 2013; Mantel et al., 2016; 2013; Dekel et al., 2021; Vimonpatranon et al., 2022), but drug-induced consequences have not yet been established. For this reason, we evaluated their role in parasite invasion and THP-1-derived macrophage stimulation. We found that EVs from CQ-treated iRBCs isolated from *Plasmodium* late stages are highly enriched in proteasome subunits and ribosomal proteins. We show that this unique protein profile is not involved in regulating the parasite growth rate. Nevertheless, it might be associated with the activation of interferon (IFN) signaling pathways mediated by IL-6 in THP-1-derived macrophages with either pro- or anti-inflammatory effects.

Our results shed new insights into a novel CQ-induced red blood cell death mechanism that targets the parasite and specific components of the infected host cell and provides an in-depth characterization of CQ’s downstream effects beyond the parasite cell.

## Results

### Chloroquine treatment triggers the externalization of phosphatidylserine and ceramide abundance in the iRBC plasma membrane

**Figure 1:**
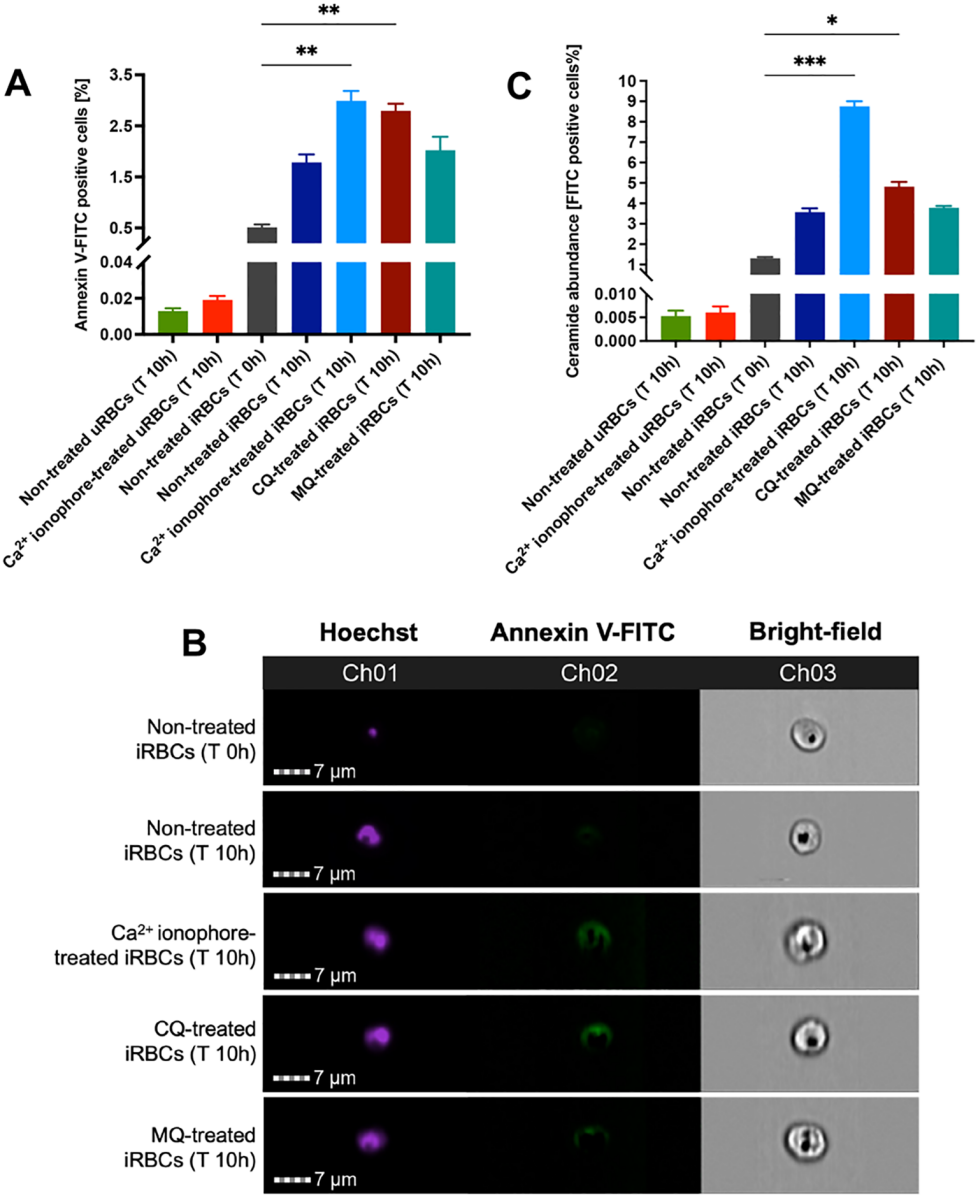
Effect of CQ on PS exposure and ceramide abundance in iRBCs. **(A)** PS exposure in iRBCs represented by the percentage of Annexin-V FITC positive cells. **(B)** Fluorescence microscopy images of PS flipping to the outer leaflet of iRBCs plasma membrane. **(C)** Ceramide abundance in iRBCs represented by the percentage of FITC-positive cells. The results correspond to the mean ± sem of 3 independent experiments; *p < 0.05, **p < 0.01, ***p < 0.001 by Kruskal-Wallis test with *post-hoc* Dunn’s test.

To determine if iRBCs expose PS after CQ treatment due to calcium redistribution, synchronized *P. falciparum* 3D7 cultures at 10% parasitemia (mid-late trophozoites) were incubated for 10h with 1 µM of Ca^2+^ ionophore (ionomycin), CQ, Mefloquine (MQ) and 1**×** PBS (non-treated iRBCs). MQ was included as a DV non-disrupting antimalarial drug (Lee et al., 2014; Tong et al., 2018) and uninfected red blood cells (uRBCs) as a negative control due to their low basal levels of PS exposure. Only Ca^2+^ ionophore-treated uRBCs and iRBCs were incubated with malaria culture media (MCM) and ringer solution (RS) to stimulate PS flipping as a positive control. Enriched schizonts by magnetic-activated cell sorting (MACS) were used for Annexin V-FITC and Hoechst staining. The results showed a modest but significant rise of PS exposure for Ca^2+^ ionophore- and CQ-treated iRBCs with 5.85 and 5.46 fold change, respectively (**Figure 1A**). Furthermore, high-content fluorescence microscopy was used to obtain images from non-treated iRBCs, Ca^2+^ ionophore-treated iRBCs and drug-treated iRBCs after 10h of incubation to show PS flipping to the outer leaflet of the plasma membrane, supporting the flow cytometry data (**Figure 1B**). As reported in the literature (Boulet et al., 2018), increased intracellular calcium in RBCs triggers the activation of platelet-activating factor (PAF) which stimulates sphingomyelinase to cleave sphingomyelin producing ceramide that accumulates in the plasma membrane. Ceramide abundance was determined by staining MACS-enriched schizonts with anti-ceramide antibody and Hoechst. The results displayed the same trend obtained by PS exposure where Ca^2+^ ionophore-treated and CQ-treated iRBCs had modest but significantly higher levels of ceramide compared to non-treated iRBCs (**Figure 1C**). These findings demonstrate that increased PS exposure and ceramide abundance are distinct eryptotic features exhibited by CQ-treated iRBCs.

### Digestive vacuole disruption by chloroquine is associated with cell shrinkage and membrane blebbing

**Figure 2:**
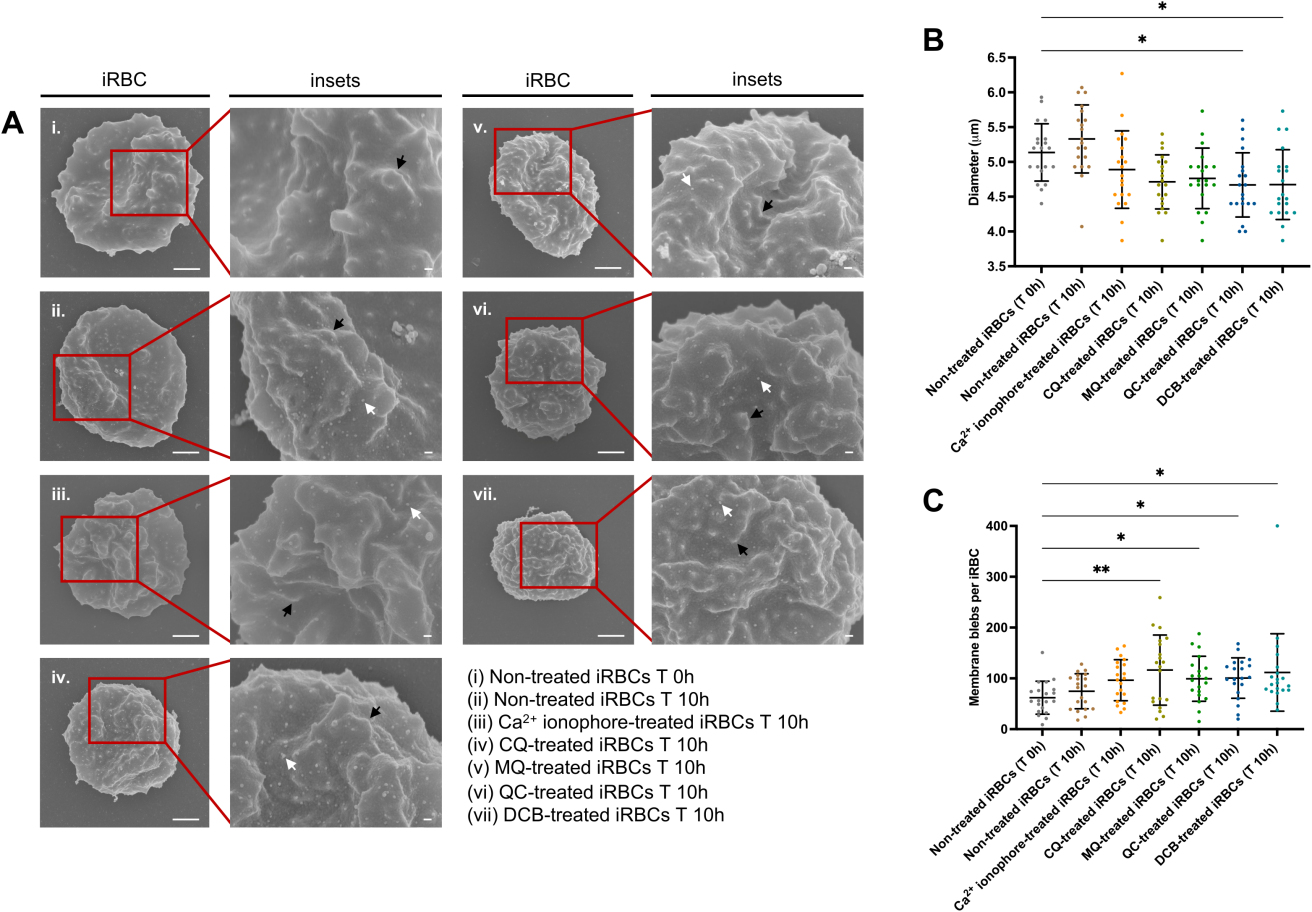
DV-disrupting compounds treatment is associated with an eryptotic morphology in iRBCs: cell shrinkage and microvesiculation. **(A)** Representative scanning electron micrographs of non-treated iRBCs T 0h **(i)**, non-treated iRBCs T 10h (ii), Ca^2+^ ionophore-treated iRBCs T 10h (iii), CQ-treated iRBCs T 10h (iv), MQ-treated iRBCs T 10h (v), QC-treated iRBCs T 10h (vi) and DCB-treated iRBCs T 10h (vii). The black arrows denote knobs and white arrows denote blebs. Scale bars of the original and zoom images represent 1 μm and 100 nm, respectively. **(B)** Diameter of iRBCs obtained from SEM images. **(C)** Number of membrane blebs on iRBCs surface quantified from SEM images. For **(B)** and **(C)**, 20 images per condition were randomly selected and used for the scatter plots. The data represent the mean ± SD; *p < 0.05, **p < 0.01 by Kruskal-Wallis test with *post-hoc* Dunn’s test.

Similar to the CQ effect on iRBCs leading to calcium homeostasis disruption and programmed cell death features of the parasite, a previous study from our laboratory revealed that this novel mode of killing could be triggered by another antimalarial drug, quinacrine (QC), with its ability to induce calcium redistribution at submicromolar levels from DV permeabilization (Lee et al., 2014). Likewise, in the same study, a Na^+^/Ca^2+^ exchanger inhibitor, 3’,4’-dichlorobenzamil (DCB) was proposed as a possible new class of DV-destabilizing agent (Lee et al., 2014). This led us to investigate if CQ, QC and DCB treatment on iRBCs result in an eryptotic morphology such as cell shrinkage and membrane blebbing. Scanning electron microscopy (SEM) was performed using magnet-purified mature parasite forms at the starting point of the treatment and after 10h (**Figure 2A**). Only QC- and DCB-treated iRBCs showed a significant reduction in the cell diameter; however, the p-value of 0.0556 for CQ-treated iRBCs approached statistical significance, suggesting that CQ treatment also results in cell shrinkage (**Figure 2B**). Moreover, the blebs on the surface of iRBCs distinguishable from the protrusions called knobs, were counted from 20 scanning electron micrographs per each condition. All drug-treated iRBCs revealed a significant increase in blebs after 10h compared to non-treated iRBCs at T 0h (**Figure 2C**). Notably, CQ induced the largest number of blebs among the compounds tested in the experiment.

### Calcium redistribution by chloroquine, quinacrine, and 3’,4’-dichlorobenzamil disrupt the iRBC cytoskeleton network

**Figure 3:**
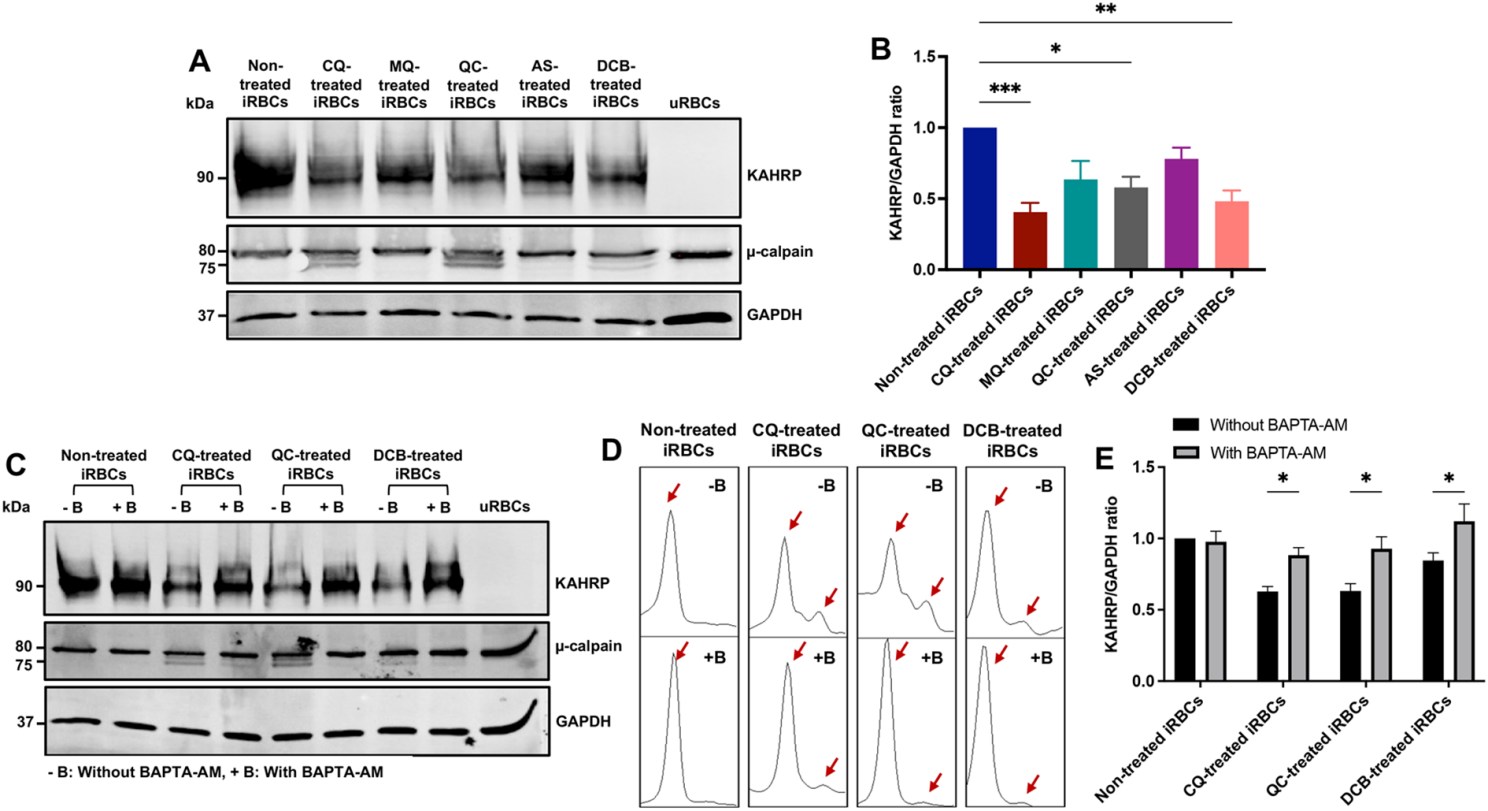
KAHRP degradation and µ-calpain activation are mediated by calcium redistribution in iRBCs treated with CQ, QC and DCB. **(A)** Western blot of KAHRP and µ-calpain protein expression in drug-treated iRBCs. **(B)** Quantification of KAHRP/GAPDH ratio normalized to non-treated iRBCs. **(C)** Western blot of KAHRP and µ-calpain protein expression in drug-treated iRBCs preincubated with BAPTA-AM. **(D)** Densitometry plots of µ-calpain bands with and without BAPTA-AM. The red arrows indicate the two μ-calpain forms observed in the blot, the 80 kDa band (full-length large subunit) and the 75 kDa band (autolytically cleaved active form). **(E)** Quantification of KAHRP/GAPDH ratio normalized to non-treated iRBCs with and without BAPTA-AM. The data represent the mean ± sem of 6 independent experiments; *p < 0.05, **p < 0.01, ***p < 0.001 by Kruskal-Wallis test with *post-hoc* Dunn’s test **(B)** and two-way ANOVA test with *post-hoc* Bonferroni’s test **(E)**.

It has been reported that an increase in intracellular calcium concentration activates calpain, a calcium-dependent cytosolic protease from the host RBC which leads to cytoskeleton cleavage (Bebawy et al., 2013). *Plasmodium* exports numerous proteins to the RBC plasma membrane throughout its development, late stages particularly, translocate the knob-associated histidine-rich protein (KAHRP) which interacts with the RBC cytoskeleton, and, together with other macromolecules, form protrusions on the cell surface known as knobs (Rug et al., 2006). To determine the effects of the antimalarial compounds as RBC cytoskeleton network disruptors, µ-calpain activation and KAHRP levels were analyzed by western blot. iRBCs at mid-late trophozoites were treated for 10h with CQ, MQ, QC, artesunate (AS) and DCB. KAHRP bands were quantified using Image J software. The DV-disrupting compounds CQ, QC, and DCB led to doublet bands for µ-calpain (**Figure 3A**). In the presence of calcium, the 80 kDa form of µ-calpain is cleaved to the 75 kDa form with increased catalytic activity. Furthermore, these compounds caused a significant loss of KAHRP, whereas the non-disrupting antimalarial drugs MQ and AS did not (**Figures 3A, B**). To confirm that calcium redistribution is the main factor for this proteolytic processing, a 30-minute pre-incubation with the calcium chelator BAPTA-AM was performed, followed by a 10-hour treatment with CQ, QC and DCB. The compounds were selected based on their significance displayed previously. The data showed that BAPTA-AM reduced μ-calpain activation in iRBCs treated with CQ, QC and DCB (**Figure 3C**). The densitometry plots of μ-calpain were obtained using Image J software to determine if the second peak, corresponding to the 75 kDa active form, is absent with BAPTA-AM (**Figure 3D**). Likewise, the calcium chelator pre-treatment demonstrated a significant rescue of KAHRP levels (**Figures 3C, E**). These findings demonstrate that calcium homeostasis disruption triggered by CQ, QC and DCB treatment in iRBCs mediates calpain activation and loss of KAHRP.

### iRBC-derived EVs quantity, distribution, and protein profile

**Figure 4:**
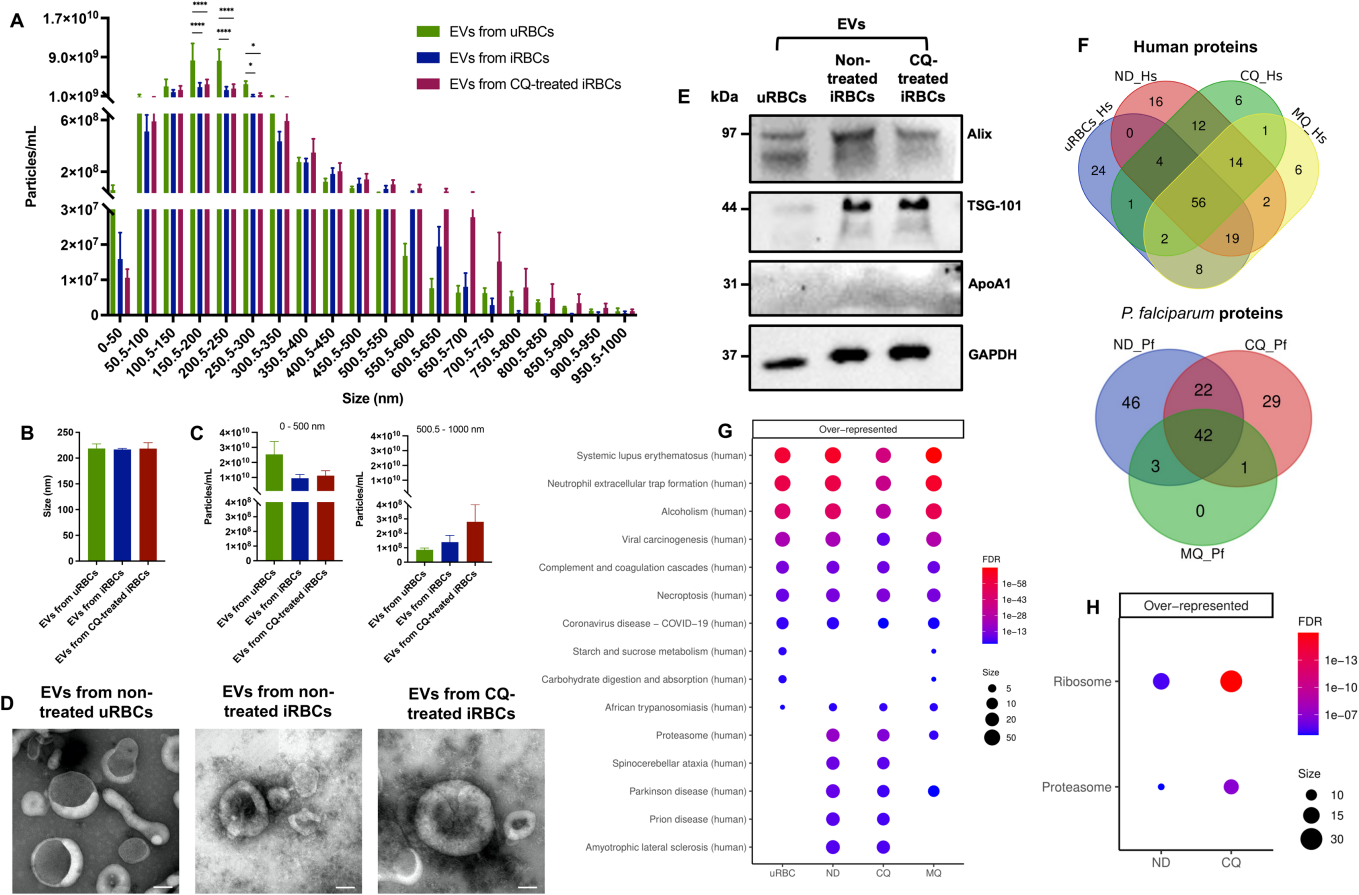
Characterization of iRBC-derived EVs. **(A)** Number of EVs from non-treated uRBCs, non-treated iRBCs, and CQ-treated iRBCs per size categories between 0 – 1–000 nm obtained by NTA. **(B)** Particle size average from NTA measurements. **(C)** Total number of particles, from 0–500 nm and 500.5 – 1–000 nm. **(D)** Representative transmission electron negative staining micrographs of EV samples (scale bars = 100 nm; original magnification at ×35 000). **(E)** Western blot of EV markers ALIX and TSG-101. ApoA1 served as a negative marker control and GAPDH as a loading control. **(F)** Venn diagrams illustrating the unique and shared human and parasite proteins identified above the detection threshold in EV samples by LC-MS. KEGG pathway enrichment analysis of **(G)** human and **(H)** parasite proteins from EV samples. The results are presented in mean ± sem of 3 independent experiments; *p < 0.05, ****p < 0.0001 by two-way ANOVA test with *post-hoc* Bonferroni’s test **(A)** and Kruskal-Wallis test with *post-hoc* Dunn’s test (B & C). uRBCs: EVs from non-treated uRBCs; ND: EVs from non-treated iRBCs; CQ: EVs from CQ-treated iRBCs; MQ: EVs from MQ-treated iRBCs.

The iRBCs-derived EVs released into the extracellular milieu are important mediators of parasite-parasite and parasite-host communication (Carrera-Bravo et al., 2021). EVs from non-treated uRBCs, non-treated iRBCs and CQ-treated iRBCs were isolated by differential ultracentrifugation. Nanoparticle tracking analysis (NTA) was employed to determine the number of EVs (particles/mL) per size range, from 0 – 1–000 nm (**Supplementary Figure S1**). EVs from uRBCs had significantly higher densities compared to EVs from non-treated and CQ-treated iRBCs in 3 size categories (150.5–200 nm, 200.5–250 nm, and 250.5–300 nm) (**Figure 4A**). On average, EVs from all samples measured 200 nm approximately (**Figure 4B**). Once the EV size was divided into two groups (0–500 nm; 500.5 – 1–000 nm) it was observed that non-treated uRBCs preferentially released smaller EVs within the size category 0–500 nm, having a mean value of 2.54 x 10^10^ particles/mL (**Figure 4C**). However, CQ-treated iRBCs secreted larger EVs within the size category 500.5 – 1–000 nm with a mean value of 2.80 x 10^8^ particles/mL (**Figure 4C**). EVs were visualized by transmission electron microscopy (TEM) negative staining, validating the cup-shaped morphology and the presence of lipid bilayer-enclosed nanosized structures (**Figure 4D**) which coincides with the average size reported by NTA. Using western blot, the EV markers ALIX and TSG-101 were found in all EV samples, while ApoA1, considered the negative control, was absent (**Figure 4E**). Liquid Chromatography Mass Spectrometry (LC-MS) analysis was performed to determine the protein profile of EVs produced under RBC’s physiological condition, parasite development and antimalarial drug treatment. EVs from MQ-treated iRBCs were used in addition to the set of samples described above. MQ was included in view of its non-disruptive properties on calcium homeostasis from DV membrane permeabilization. Among the proteins identified above the detection threshold, 56 human and 42 parasite proteins were common in all samples (**Figure 4F**). Additionally, unique proteins were identified for each sample. Interestingly, the Kyoto Encyclopedia of Genes and Genomes (KEGG) pathway analysis revealed an over representation of human proteasomal proteins in EVs from non-treated, CQ-treated and MQ-treated iRBCs (**Figure 4G**), whereas *Plasmodium* proteasome and ribosome were only significantly enriched in EVs from non-treated iRBCs and EVs from CQ-treated iRBCs (**Figure 4H**). These results suggest that parasite development along with drug pressure play a role in the size and protein composition of EVs released by iRBCs.

### Chloroquine treatment leads to an enrichment of proteasome subunits and ribosomal proteins in iRBC-derived EVs

**Figure 5:**
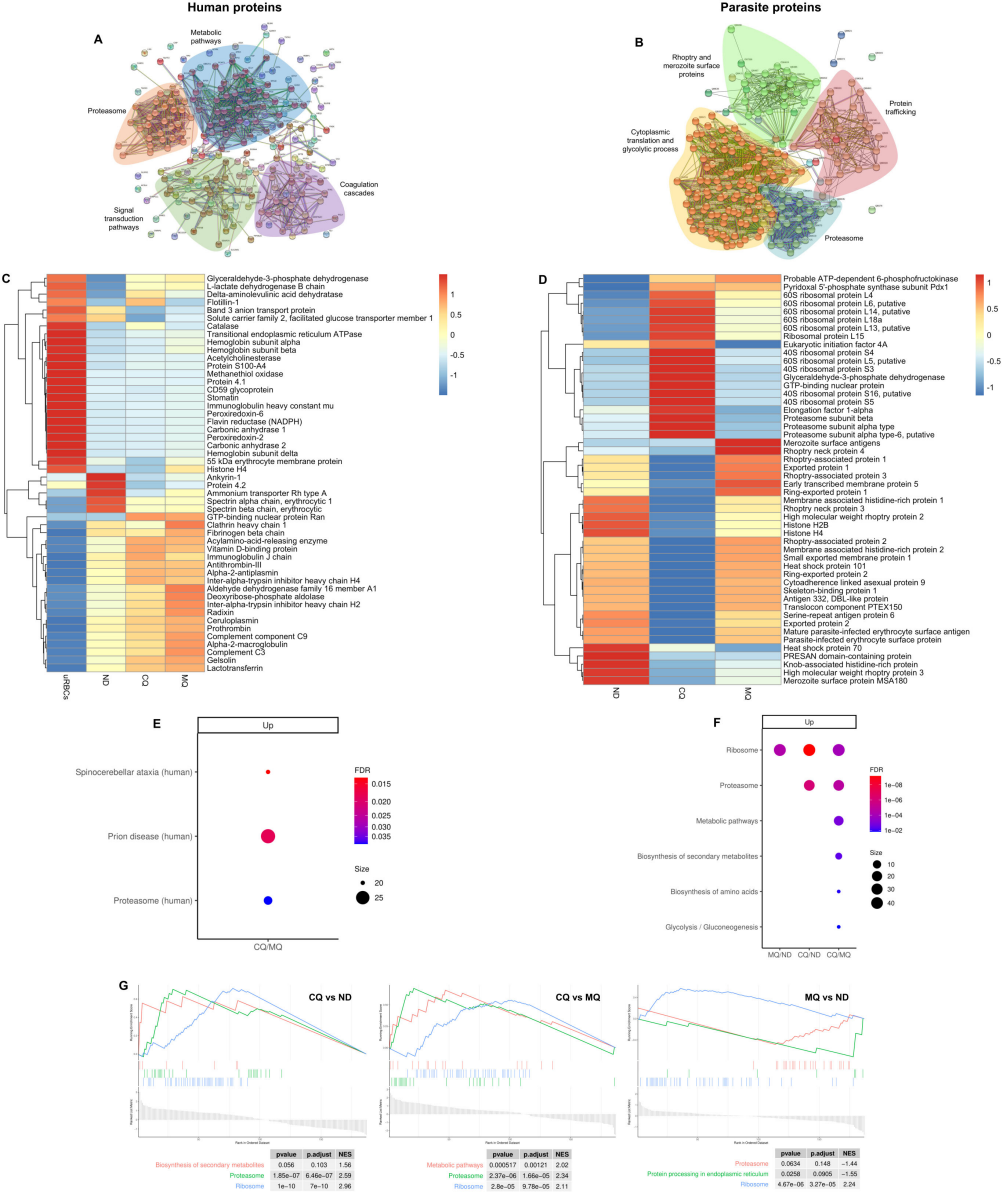
Quantitative proteomic data of EVs. **(A)** Human and **(B)** parasite protein interaction networks of EV samples. Whole proteins identified from both datasets were used. Protein nodes and interactions were colored according to MCL clustering using STRING software. Heatmap of the 50 most abundant **(C)** human and **(D)** *P. falciparum* proteins. The average of 3 independent experiments per sample is shown. The colors represent Z score mean protein expression values for samples. KEGG enrichment analysis of **(E)** human and **(F)** parasite proteins. Bubble size: Number of proteins assigned to the pathway; Color: adjusted p-value. **(G)** GSEA analysis of the top 3 positively and negatively enriched KEGG pathways from parasite proteins. uRBCs: EVs from non-treated uRBCs; ND: EVs from non-treated iRBCs; CQ: EVs from CQ-treated iRBCs; MQ: EVs from MQ-treated iRBCs.

In order to quantify the differential abundance of EV proteins in response to antimalarial drug treatment, we used Sequential Window Acquisition of all Theoretical Mass Spectra (SWATH-MS) analysis. The statistical cut-off criteria were minimum two unique peptides quantified per protein, fold change ≥ 1.5 and adjusted p-value ≤ 0.05. Moreover, STRING analysis for protein-protein interaction networks highlighted the 4 main clusters of the human (**Figure 5A**) and *Plasmodium* (**Figure 5B**) proteome found in our EV samples. Intriguingly, the proteasome protein cluster was represented in both human and *Plasmodium* datasets. The relative abundance of the 50 most abundant proteins of each dataset was visualized in heatmaps. For example, hemoglobin subunits alpha and beta were highly enriched as expected in EVs from non-treated uRBCs due to the absence of parasite invasion (**Figure 5C**). Meanwhile, ribosomal proteins and proteasome subunits were significantly enriched in EVs from CQ-treated iRBCs compared to EVs from non-treated and EVs from MQ-treated iRBCs (**Figure 5D**). Gene ontology (GO) analysis was performed to provide functional insights of the EV protein cargo. For human proteins, no pathways associated with the proteasome complex were found in cellular component, biological process, and molecular function (**Supplementary Figures S2A–C**). On the other hand, parasite proteins, proteasome core complex and proteasome alpha-subunit complex were highly enriched cellular components in EVs from CQ-treated iRBCs (**Supplementary Figure S2D**, **Supplementary Table S1**). KEGG analysis determined the enriched pathways in both datasets. The proteasome pathway was upregulated for human (**Figure 5E**) and parasite (**Figure 5F**) proteins in EVs from CQ-treated iRBCs, whereas the parasite ribosome pathway (**Figure 5F**) was upregulated in EVs from CQ-treated and MQ-treated iRBCs (**Supplementary Table S1**). GSEA plots of the top 3 significant KEGG pathways from parasite proteins in EVs from CQ-treated iRBCs and EVs from MQ-treated iRBCs compared with each other and to EVs from non-treated iRBCs are shown in **Figure 5G**. Similarly, cell lysates were assessed by SWATH-MS where GO and KEGG analysis indicated human proteasome-related pathways (**Supplementary Figures S3A, B**) and parasite ribosome-related pathways (**Supplementary Figures S3C, D**) to be upregulated in CQ-treated iRBCs in contrast with non-treated iRBCs. These results indicate that proteasome and ribosome protein clusters are characteristic phenotypes of CQ-treated iRBCs and their EVs.

**Figure 6:**
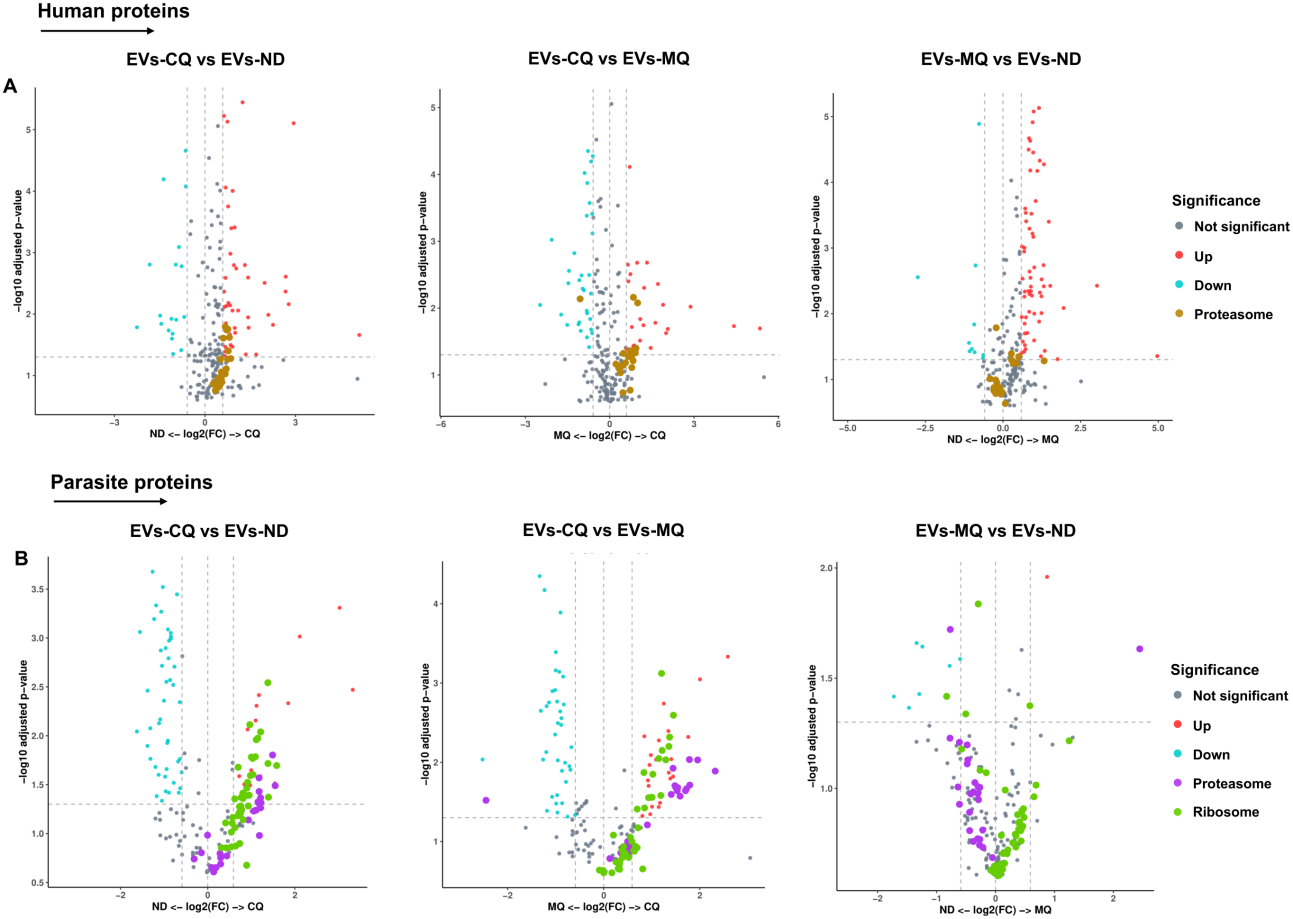
Significantly enriched proteasome and ribosomal proteins in iRBC-derived EVs. **(A)** Volcano plots of differentially expressed human proteins in iRBC-derived EVs. Proteasome subunits (brown dots) are highlighted. **(B)** Volcano plots of differentially expressed *P. falciparum* proteins in iRBC-derived EVs. Proteasome subunits (purple dots) and ribosomal proteins (green dots) are highlighted. X-axis, vertical-dotted lines: log2(FC) cut-off is 0.585, corresponding to fold change = 1.5. Y-axis, horizontal-dotted line: Significance cut-off, adjusted p-value = 0.05 (A & B). EVs-ND: EVs from non-treated iRBCs; EVs-CQ: EVs from CQ-treated iRBCs; EVs-MQ: EVs from MQ-treated iRBCs.

Differentially expressed proteins from both datasets were indicated in volcano plots. 6 human proteasomal subunits were upregulated in EVs from CQ-treated iRBCs compared to EVs from non-treated and EVs from MQ-treated iRBCs (**Figure 6A**, **Supplementary Table S2**). 8 and 13 parasite proteasome subunits were upregulated in EVs from CQ-treated iRBCs compared to EVs from non-treated and MQ-treated iRBCs, respectively (**Figure 6B**; **Supplementary Table S3**). Moreover, 23 and 16 parasite ribosomal proteins were upregulated in EVs from CQ-treated iRBCs compared to EVs from non-treated and EVs from MQ-treated iRBCs, respectively (**Figure 6B**; **Supplementary Table S3**). From this data, we conclude that CQ is capable of triggering DV permeabilization-induced calcium dysregulation leading to the release of EVs with a unique cargo.

### The role of EVs from CQ-treated iRBCs in parasite invasion and THP-1-derived macrophage response

Our results for LC-MS and SWATH-MS demonstrated a unique protein profile composed of highly enriched ribosomal proteins and proteasome subunits in EVs from CQ-treated iRBCs which might play an essential role in recipient cells. Firstly, the chymotrypsin-like activity of the proteasome complexes in EVs was determined, showing that the proteolytic activity was highly significant in the proteasome complexes of iRBC-derived EVs (**Supplementary Figure S4**). Subsequently, we investigated their effect on the parasite growth rate of *P. falciparum* cultures. A 32-hour invasion assay was performed with Cell Tracker Deep Red (CTDR)-labeled uRBCs, MACS-enriched schizonts, and EVs from non-treated uRBCs, EVs from non-treated iRBCs, EVs from CQ-treated iRBCs and 1**×** PBS (control). Giemsa smears were prepared to determine the parasitemia by light microscopy (**Supplementary Figure S5A**), whereas the DNA-specific dye Hoechst was used to determine parasite invasion by flow cytometry. No significant difference in the parasite growth rate was found among EV samples compared to the control for both methods (**Supplementary Figure S5B**). This result suggests that the high content of proteasomal subunits and ribosomal proteins in EVs from CQ-treated iRBCs did not play a role in the invasion efficiency or higher concentrations of EVs are needed.

**Figure 7:**
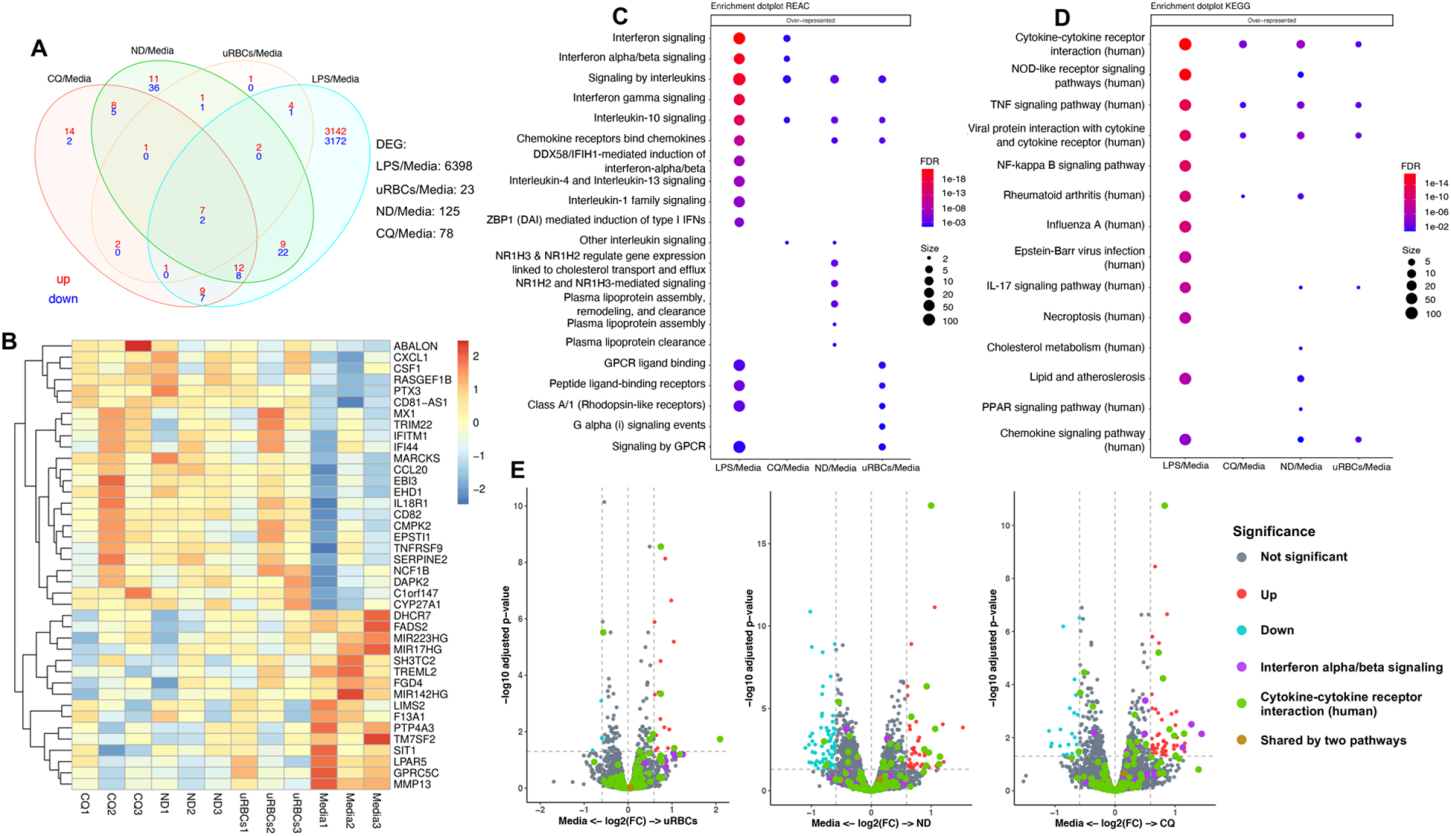
RNA sequencing data of THP-1-derived macrophages upon stimulation with EVs. **(A)** Venn diagram of significant DEGs. Upregulated and downregulated genes are shown in red and blue, respectively. **(B)** Heatmap of top 40 DEG from THP-1-derived macrophages stimulated with EVs from CQ-treated iRBCs. The colors represent Z score mean protein expression values for samples. Over-representation of **(C)** Reactome and **(D)** KEGG pathway analysis. Bubble size: Number of genes assigned to a pathway; Color: adjusted p-value. **(E)** Volcano plots denote the upregulated and downregulated genes of Reactome (purple dots) and KEGG (green dots) pathways. X-axis, vertical-dotted lines: log2(FC) cut-off is 0.585, corresponding to fold change = 1.5. Y-axis, horizontal-dotted line: Significance cut-off, adjusted p-value = 0.05. uRBCs: EVs from non-treated uRBCs; ND: EVs from non-treated iRBCs; CQ: EVs from CQ-treated iRBCs.

The effect of EVs on the host’s innate immune response was studied *in vitro* using THP-1-derived macrophages. THP-1 cells were differentiated with phorbol 12-myristate 13-acetate (PMA) into macrophages and stimulated with EVs from non-treated uRBCs, EVs from non-treated iRBCs and EVs from CQ-treated iRBCs for 14h. LPS and media were used as positive and negative controls, respectively. The cell culture supernatant was collected for ELISA measurement of Tumor Necrosis Factor-alpha (TNF-α), Interleukin-6 (IL-6) and Interleukin-1 beta (IL-1β) levels, and cells were collected for transcriptomic analysis. EVs from CQ-treated iRBCs significantly induced IL-6 production although the effect was not as strong as LPS (**Supplementary Figure S6**). Meanwhile, TNF-α and IL-1β were not significantly changed. Transcriptomic profiling showed that EVs from non-treated uRBCs, EVs from non-treated iRBCs and EVs from CQ-treated iRBCs induced 23, 125 and 78 differentially expressed genes (DEG), respectively (**Figure 7A**). The top 40 DEGs for EVs from CQ-treated iRBCs against media were displayed in a heatmap (**Figure 7B**), revealing some genes with similar relative expression in cells stimulated with EV samples. Interestingly, the Reactome pathway analysis indicated interleukin pathways as over-represented among DEGs for THP-1-derived macrophages stimulated with EV samples (**Figure 7C**). Whereas interferon signaling and interferon alpha/beta signaling were uniquely over-represented pathways upon stimulation with EVs from CQ-treated iRBCs. For KEGG analysis, TNF-α signaling pathway, cytokine-cytokine receptor interaction and viral protein interaction with cytokine and cytokine receptor were over-represented pathways for THP-1-derived macrophages stimulated with EV samples (**Figure 7D**). Additionally, GO analysis disclosed biological process pathways associated to viral life cycle for THP-1-derived macrophages stimulated with EVs from non-treated iRBCs and EVs from CQ-treated iRBCs (**Supplementary Figure S7B**). DEGs of two pathways were featured in volcano plots (**Figure 7E**). The interferon alpha/beta signaling from Reactome pathways exhibited 3 interferon stimulated genes (ISG) upregulated only for EVs from CQ-treated iRBCs, while cytokine-cytokine receptor interaction from KEGG pathways displayed 4, 11 and 8 DEGs for THP-1-derived macrophages stimulated with EVs from non-treated uRBCs, EVs from non-treated iRBCs and EVs from CQ-treated iRBCs, respectively (**Supplementary Table S4**).

## Discussion

Our previous findings demonstrated that CQ doses ≤3 µM selectively permeabilize the *Plasmodium* DV and mitochondria membranes, triggering calcium redistribution in the iRBC *in vitro* and *in vivo*, without causing generalized toxicity and maintaining clinical relevance (Ch’ng et al., 2011, 2014). Here, we present evidence that 1 µM CQ-induced calcium homeostasis disruption is associated with eryptotic hallmarks in the host RBC and a distinct EV proteomic profile.

We assessed the impact of CQ treatment on mid-late trophozoites iRBCs and observed increased PS exposure. CQ’s effect on intracellular calcium dysregulation may activate calcium-dependent scramblases, leading to the outward translocation of PS in the plasma membrane. Additionally, literature indicates that elevated intraerythrocytic calcium levels are associated with increased platelet-activating factor (PAF) levels. PAF stimulates sphingomyelinase, which cleaves sphingomyelin to produce ceramide (Boulet et al., 2018; Lang et al., 2005). This ceramide accumulation in the plasma membrane results in lipid scrambling and PS exposure (Lang et al., 2015, 2004). Our data support these findings, demonstrating a significant increase in ceramide levels in CQ-treated iRBCs.

Previously, our group demonstrated that calcium redistribution induced by CQ, QC and DCB leads to programmed cell death features in parasite cultures, such as mitochondrial depolarization and DNA loss (Lee et al., 2014). However, research on the effects of these compounds on host RBCs and their downstream consequences was lacking. As reported in the literature (Boulet et al., 2018; Bookchin et al., 1987), an increase in intracellular calcium activates calcium-sensitive potassium channels (Gardos channels) which prompt iRBC membrane hyperpolarization through potassium and chlorine efflux, leading to osmotic dehydration and cell shrinkage. Our current findings showed that CQ, QC and DCB induce cell shrinkage in iRBCs. SEM images revealed numerous blebs on the surface of drug-treated iRBCs, with the highest abundance observed in CQ-treated iRBCs. This was confirmed by the activation of the calcium-dependent protease µ-calpain, which cleaves the RBC cytoskeleton, leading to protein network disruption in the plasma membrane following CQ, QC and DCB treatment. Wieschhaus et al. reported that µ-calpain negatively regulates RBC deformability and filterability without visibly affecting RBC lifespan under physiological conditions (Wieschhaus et al., 2012). Using a µ-calpain knockout mouse model, they demonstrated that this protease plays a direct role in RBC shape regulation and the shape transition rate in a calcium-dependent manner (Wieschhaus et al., 2012). Consistent with these findings, our data suggest that unregulated µ-calpain contributes to the disruption of cytoskeletal and membrane integrity in iRBCs. Moreover, western blot experiments displayed reduced levels of KAHRP with µ-calpain activation. However, BAPTA-AM-mediated rescue of KAHRP, combined with a decrease in the µ-calpain catalytic subunit, supports the role of calcium in the degradation process. This indicates that membrane blebbing is associated with the catastrophic events resulting from calcium homeostasis disruption by CQ, QC and DCB treatments in iRBCs.

Following the downstream effects of CQ treatment on the cell, we characterized iRBCs-derived EVs to explore their phenotype. It has been demonstrated by electron microscopy that malaria EVs have an average diameter of 200 nm with an intact lipid bilayer (Regev-Rudzki et al., 2013; Mantel et al., 2013; Babatunde et al., 2018; Blow and Buck, 2022; Dekel et al., 2021). Likewise, our TEM and NTA data agreed with these studies. Interestingly, the predominance of larger EVs from CQ-treated iRBCs suggests that the cell could be undergoing eryptosis and releasing eryptotic bodies (Kučuk et al., 2021). Proteomic profiling and significant markers from EVs were determined, showing similarities between our protein dataset and the iRBC-derived EV proteins reported by previous studies (Mantel et al., 2013; Dekel et al., 2021). Therefore, we propose that the variability in malaria EV characterization regarding morphology, size, concentration and cargo may be attributed to factors including parasite stage, culture conditions, EVs isolation methods, and the solution used to resuspend isolated EVs.

KEGG pathway analysis of LC-MS datasets revealed the presence of parasite ribosomal proteins, along with human and parasite proteasome subunits in EVs from non-treated and CQ-treated iRBCs. Subsequently, using SWATH-MS quantitative proteomics, we identified the proteasome as one of the four major clusters for human and parasite proteins within the EVs. The detection of proteasome subunits in EVs aligns with previous studies from other protozoa, helminths and ectoparasites (Sharon and Regev-Rudzki, 2021). In the protozoan *Acanthamoeba castellanii*, the EV-associated proteasome regulates encystation and extracellular proteolytic activities (Lin et al., 2019), whereas in *Trichinella* sp*iralis*, the EV-associated proteasome subunit beta type-7 (PST) is expressed at different developmental stages and confers protective immunity against parasite infection in BALB/c mice immunized with the recombinant PST (Yang et al., 2015; Gao et al., 2022). In support with these findings, Dekel et al. demonstrated that iRBC-derived EVs contain functional 20S proteasome complexes (Dekel et al., 2021). Our quantitative proteomic data further reinforce this and, for the first time, show the upregulation of human and parasite 20S proteasome subunits, as well as human 26S proteasome subunits, in EVs from CQ-treated iRBCs. *Plasmodium* 40S and 60S ribosomal proteins were also upregulated in these EVs, potentially reflecting a disturbance in ribosome biogenesis caused by CQ treatment. In agreement with existing literature, chemical agents, lack of nutrients and gene deregulation can trigger ribosomal stress, leading to the accumulation of ribosome-free ribosomal proteins (Zhou et al., 2015). This disruption may account for the increased presence of ribosomal proteins in EVs from CQ-treated iRBCs. Among these, *P. falciparum* 60S acidic ribosomal protein P2 (P2) was particularly abundant. Studies in murine models have shown that P2 has strong immunogenic potential, inducing IgG titers comparable to the parasite-specific antigen Msp-1_19_ (Szuster-Ciesielska et al., 2019). Moreover, P2 has been localized on the iRBC surface during late trophozoite/early schizont stages, and blocking its accessibility with monoclonal antibodies arrests nuclear division (Das et al., 2021). In this context, P2 together with other ribosomal proteins may have extra-ribosomal roles and contribute as immunogenic antigens during malaria pathogenesis. Our mass spectrometry data describes the detrimental effects of CQ in iRBCs and indicates that calcium homeostasis disruption may serve as an internal stimulus for downstream mechanisms, promoting the degradation of proteasome and ribosome complexes.

Active 20S proteasome complexes along with kinases delivered by iRBC-derived EVs to uRBCs were shown to remodel the cytoskeleton and alter membrane stiffness, facilitating parasite invasion (Dekel et al., 2021). Unlike this finding, we did not observe a significant change in the parasite growth in response to EVs. In contrast, Vimonpatranon et al. tested EVs from 3D7 and three other *P. falciparum* strains, finding that only 3D7 showed no significant effect on invasion efficiency, with no proteasome detected in the EV cargo (Vimonpatranon et al., 2022). These discrepancies may be due to parasite stage-dependent EV cargo, as both studies used trophozoites. We collected EVs after a 10-hour incubation, starting from mid-late trophozoites (36 – 38h p.i.) to schizonts (46 – 48h p.i) and calculated EV concentration based on protein yield (300 μg, ~4 x 10^9^ particles/mL). In contrast, the other studies based EV concentration on particle count alone (Dekel et al., 2021; Vimonpatranon et al., 2022). These findings highlight that the role of EVs in parasite invasion may depend on both cargo and particle concentration.

As mentioned before, parasite molecules within EVs vary depending on parasite developmental stages which might differentially impact the immune system. Monocytes, macrophages and NK cells have been shown to participate in immunomodulation in response to iRBC-derived EVs from ring and trophozoite stages (Mantel et al., 2013; Dekel et al., 2021; Vimonpatranon et al., 2022; Ye et al., 2018; Ofir-Birin et al., 2021). In our current study, we focused on the effects of EVs from mid-late trophozoites to schizonts drug-treated iRBCs in the gene expression and cytokine profile of THP-1-derived macrophages. We observed a modest but significant release of IL-6 in response to EVs from CQ-treated iRBCs. While IL-6 has been reported to induce ISGF3γ expression, a component of the ISGF3 complex involved in type I IFN responses (Weihua et al., 2000), we did not directly assess its expression in this study. Notably, we found that the Reactome pathway of interferon alpha/beta signaling, represented by ISGs (MX1, IFIT1, and IFITM1) was over-represented after stimulation with EVs from CQ-treated iRBCs (**Supplementary Table S4**). This observation might hint at a potential IL-6-mediated modulation of IFN signaling; however, we emphasize that this remains a speculative mechanism and requires further investigation. Alternatively, IL-6-independent pathways or direct effects of EV cargo could underlie the observed ISG expression.

**Figure 8:**
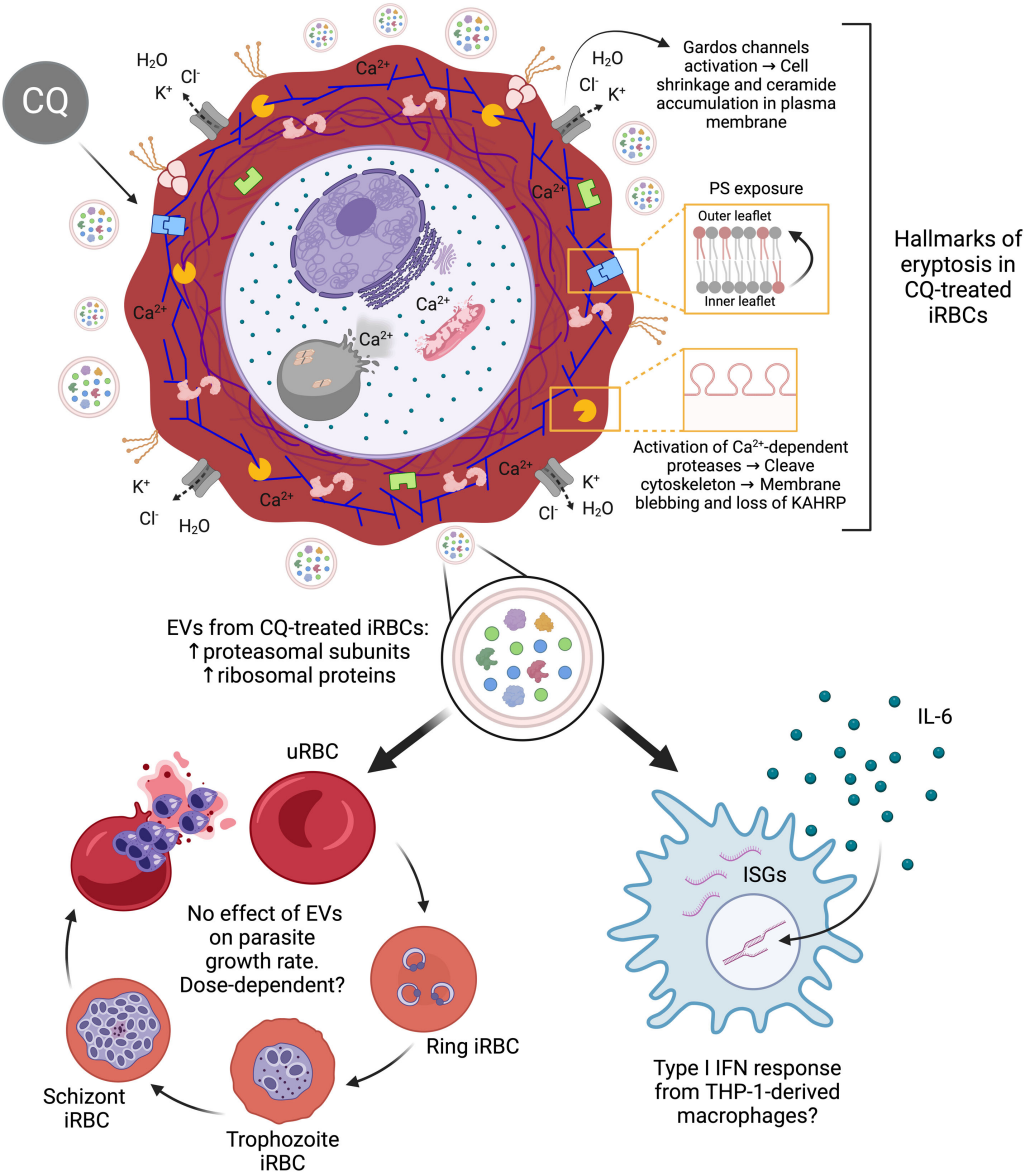
Proposed model of downstream effects triggered by CQ treatment in iRBCs. The diagram was created based on the results observed in this study associated with intracellular calcium homeostasis dysregulation in CQ-treated iRBCs. *Created with* *BioRender.com* .

Further studies using EV multi-omics and timepoint multiplex cytokine assays are needed to determine how drug pressure influences EV cargo profiles and whether EVs from CQ-treated iRBCs elicit a pro-inflammatory or anti-inflammatory type I IFN response in a time-dependent manner. In conclusion, we reveal that CQ-induced calcium redistribution in iRBCs triggers eryptotic hallmarks in host RBCs, leading to a rapid killing effect, with downstream events that may have significant implications for parasite pathogenicity (**Figure 8**).

## Materials and methods

### *Plasmodium falciparum* culture

3D7 *P. falciparum* laboratory strain (MRA-104; MR4, ATCC Manassas Virginia) was synchronized for two cycles prior to the start of the experiments. The cultures were maintained at a relatively high parasitemia level of 10% (mid-late trophozoites) at 2.5% hematocrit. The malaria culture medium (MCM) comprised a filter-sterilized mixture of RPMI 1640 (Life Technologies, USA), 2.2 g/L sodium bicarbonate, 0.5% Albumax II (Gibco, Thermo Fisher Scientific, USA), 0.005% hypoxanthine (Sigma-Aldrich, USA), 0.03% L-glutamine (Sigma-Aldrich, USA), and 25 μg/L gentamicin (Gibco, Thermo Fisher Scientific, USA) with a final pH of 7.4. EV-depleted Albumax II MCM was used exclusively for malaria cultures intended for EV isolation. 0.5% Albumax II was prepared in RPMI 1640 and centrifuged at 150 000 × g for 2h at 4°C. EV-depleted Albumax II was collected by filtering the supernatant through a 0.22 μm filter. 100 mL of EV-depleted Albumax II was added to 900 mL of incomplete MCM which had the same concentrations as described above of RPMI 1640, sodium bicarbonate, hypoxanthine, L-glutamine and gentamicin. Type O-positive human RBCs in citrate-phosphate-dextrose with adenine (CPDA-1) anticoagulant were obtained from the Interstate Blood Bank (Memphis, TN, USA) and tested negative for infectious agents according to FDA guidelines. Parasite cultures were incubated under a gas mixture of 5% CO_2_, 3% O_2_, and 92% N_2_ at 37°C in the dark. Parasitemia and stage of the culture were assessed by light microscopy after staining methanol-fixed thin films with a diluted Giemsa stain. Parasites were synchronized to select ring-stage parasites with 5% sorbitol (Merck, Germany) at 37°C for 15 min. *P. falciparum* cultures were tested for mycoplasma once a month using MycoStrip™ (InvivoGen, USA).

### Solutions and drug preparation

For all experiments, *P. falciparum* mid-late trophozoites iRBCs cultures were treated for 10h with a final concentration of 1 μM of drug or 1× PBS (control) while uRBCs cultures were treated with 1× PBS. Drug compounds (CQ diphosphate, MQ hydrochloride, QC dihydrochloride, AS and DCB hydrochloride) and ionomycin were purchased from Sigma-Aldrich. CQ was diluted in sterile 1× phosphate-buffered saline (PBS), while the remaining drugs and ionomycin were dissolved in sterile dimethyl sulfoxide (DMSO) to stock aliquots of 10 mM at -20°C. Before any experiment, fresh working drug concentrations were prepared after dilution with sterile 1× PBS to ensure a final assay mixture of less than 0.5% DMSO. For annexin V, ceramide and SEM experiments, iRBCs and uRBCs cultures used as positive controls were incubated with the calcium ionophore ionomycin at 2.5% hematocrit in MCM and ringer solution (RS) in a 1:1 ratio. RS was prepared with 125 mM NaCl, 5 mM KCl, 1 mM MgSO_4_, 32 mM N-2-hydroxyethylpiperazine-N-2-ethanesulfonic acid (HEPES), 5 mM glucose, and 1 mM CaCl_2_; the pH was adjusted to 7.4 and the temperature kept at 37°C.

### Magnetic isolation of *P. falciparum* schizont stage iRBCs

After the 10-hour drug treatment, *P. falciparum* schizont stage iRBC cultures were enriched with the QuadraMACS Separator and LD columns (Miltenyi Biotec, Germany) and used for annexin V, ceramide, SEM, and western blot of cell lysates experiments. The LD column was washed with 1× PBS until the effluent ran clear, and the contents were eluted into a 15 mL tube.

### Annexin V-binding to determine phosphatidylserine exposure

Schizont stage iRBCs were stained with Annexin V-FITC (BioVision Inc, USA) in RS at room temperature for 30 min in the dark. The pellet was stained with Hoechst 33342 (Sigma-Aldrich, USA) for 10 min at a final concentration of 1 μg/ml in the dark. 1× PBS was used to wash the cells twice after each staining step. Annexin V-FITC and Hoechst 33342 fluorescence intensity were measured on a BD LSR Fortessa X-20 (BD, USA).

### Ceramide abundance at RBC surface

Schizont stage iRBCs were stained with 1 μg/mL ceramide monoclonal antibody (clone MID 15B4) (Enzo Life Sciences, Germany) diluted 1:10 in RS containing 0.1% bovine serum albumin (BSA) for 1h at 37°C and then washed twice using RS-BSA 0.1%. After that, cells were stained for 30 min with polyclonal fluorescein-isothiocyanate (FITC)-conjugated goat anti-mouse IgG H&L specific antibody (Abcam, UK) diluted 1:50 in RS-BSA 0.1%. The unbound secondary antibody was removed by repeated washing with RS-BSA 0.1%. The pellet was stained with Hoechst 33342 (Sigma-Aldrich, USA) for 10 min at a final concentration of 1 μg/ml in the dark followed by repeated washing with 1× PBS. FITC and Hoechst 33342 fluorescence intensity were measured on a BD LSR Fortessa X-20 (BD, USA).

### Scanning electron microscopy

MAC-sorted iRBCs coated on poly-lysine (Sigma-Aldrich, USA) glass coverslips were fixed in 2.5% glutaraldehyde, washed, treated with 1% osmium tetroxide (Ted Pella) dehydrated with increasing ethanol concentrations and critical point drying (CPD 030, Bal-Tec). Glass coverslips were sputter-coated with gold in a high-vacuum sputtering device (SCD005 sputter coater, Bal-Tec) and imaged with a field emission scanning electron microscope (JSM-6701F FEG SEM, JEOL) at an acceleration voltage of 8 kV.

### Measurement of RBCs diameter and bleb count on RBC surface

Twenty scanning electron micrographs of iRBCs per condition were randomly selected to measure the cell diameter following the scale bar of 1 μm. Additionally, the blebs on the iRBCs surface were counted using ImageJ 1.51k software to determine the drug effect on membrane blebbing.

### EV isolation

Briefly, 300 mL of *P. falciparum* schizont stage iRBC culture was harvested at 2 000 × g for 5 min using the centrifuge 5810 R (Eppendorf, Germany) to remove any additional pellet containing uninfected and iRBCs. The supernatant was centrifuged at 3–500 x g for 15 min at 4°C twice, followed by high-speed centrifugation at 15–000 x g for 30 min at 4°C using the Avanti J-26 (Beckman Coulter, USA). 300 mL of supernatants were concentrated down by the Amicon^®^ Ultra Centrifugal Filter, 50 kDa MWCO (Merck, Germany) to decrease the volume by 10 times. The concentrated supernatant was ultracentrifuged at 150–000 x g for 4h at 4°C using a SW 41 Ti rotor in a Beckman OPTIMA90X (Beckman Coulter, USA). The supernatant was discarded and the EV pellet was washed once in 12 mL of sterile 1× PBS by ultracentrifugation at 150–000 x g for 2h at 4°C. The EV pellet for each condition was resuspended in either 100 μL or 200 μL of sterile 1× PBS, depending on the characterization method and cell-based assays to be performed.

### Western blot assays

The bicinchoninic acid (BCA) assay kit (Thermo Fisher Scientific, USA) was used to determine the amount of total protein per sample, 80 μg for cell lysates and 40 μg for EVs which were loaded on SDS-PAGE gel and transferred to nitrocellulose membranes. The primary antibodies used for detection were mouse monoclonal anti-μ-calpain (Sigma-Aldrich, USA) 1:1000, mouse monoclonal anti-KAHRP clone 18.2 (European Malaria Reagent Repository, UK) 1:500, anti-TSG101 (Abcam, UK) 1:2000, anti-ALIX (Abcam, UK) 1:2000, anti-ApoA1 (Abcam, UK) 1:1000, and mouse monoclonal anti-GAPDH (Abcam, UK) 1:5000. The secondary antibody was IRDye^®^ 680RD goat anti-mouse IgG (H+L) (LICORbio, USA) 1:5000. Western blot experiments were repeated 6 times. Image collection was performed using the ChemiDoc MP Imaging System (Bio-Rad, USA). Quantification of KAHRP blots and μ-calpain densitometry plots was done using ImageJ 1.51k software. To assess that KAHRP degradation and µ-calpain activation are mediated by calcium redistribution in CQ-, QC- and DCB-treated iRBCs, a pretreatment with the calcium chelator BAPTA-AM (Abcam, UK) at 50 μM was performed by incubating the parasite cultures at 37°C for 30 min, after which the drugs were added without washing the cells following the protocol described previously.

### Nanoparticle tracking analysis

The number size distribution and concentration of EVs were analyzed using the NanoSight NS300 (Malvern Instruments, UK). The analysis is based on Brownian motion detected through the light scattering signal. For each sample, five captures were recorded, with each capture lasting 60 seconds and using a green laser at a wavelength of 532 nm. The camera level was set to 13, and the gain was adjusted to 1 to optimize particle detection. Samples were diluted 1:1000 in sterile 1× PBS. The NanoSight measurements were expressed as particles per milliliter (particles/mL).

### Transmission electron microscopy negative staining

EV pellets obtained by ultracentrifugation were resuspended with 100 μL of sterile 1× PBS. Samples were fixed with 2.5% glutaraldehyde for 1h at 4°C. A volume of 20 μL of each sample was incubated onto a Formvar Film 200 mesh, CU, FF200-Cu grid for 30 min. Negative staining was performed with 5% gadolinium triacetate for 1 min. Samples were viewed using FEI TECNAI SPIRIT G2 room temperature transmission electron microscopy. (FEI company) across ×35–000 magnification.

### Mass spectrometry data acquisition

*Liquid chromatography - mass* sp*ectrometry*. EVs were resuspended in a lysis buffer containing RIPA and protein inhibitor and mixed thoroughly. The samples were incubated for 20 min at 4°C, vortex for 15 sec, and stored at -80°C until used. EV lysate samples were processed using the S-Trap micro column (Protifi, USA) according to the manufacturer’s recommendations. Total peptide quantification was performed using the Pierce Quantitative Colorimetric Peptide Assay (Thermo Fisher Scientific, USA) to normalize sample peptide concentrations for LCMS analysis. Online reversed-phase (RP) separation of the reconstituted peptides was analyzed on the Eksigent Ekspert NanoLC 425 mass spectrometer. Solvent A for RP was 2% acetonitrile, 0.1% formic acid, while solvent B was 98% acetonitrile, 0.1% formic acid. The peptides were first trapped on a Trajan ProteoCol C18P precolumn (3 µm 120 Å, 300 µm x 10 mm) and then resolved on an Acclaim PepMap 100 C18 analytical column (3 μm 100 Å, 75 μm x 250 mm). Peptide elution was performed using a two-step linear gradient, comprising 5 - 12% solvent B over 20 min, followed by 12 - 30% solvent B over 40 min at a flow rate of 300 nL/min. Eluted peptides were directly injected into the TripleTOF 6600 system (SCIEX, USA) for MS-analysis using the information-dependent acquisition (IDA) mode (IonSpray voltage floating 2–300 V, interface heater temperature 150 C). Precursor ions were selected across an ion mass of 350 – 1–250 m/z with 250 msec accumulation time per spectrum. Maximum 20 precursors were selected for fragmentation, charge state 2-4, intensity > 125 cps with dynamic exclusion for 12 sec after first occurrence and rolling collision energy. MS/MS analysis was performed in high sensitivity mode at 100 msec accumulation time across a mass range of 100 – 1–800 m/z.

*Sequential window acquisition of all theoretical mass* sp*ectra.* For data acquisition, online RP analyses were done as described for LC-MS. The eluted peptides were injected into a TripleTOF 6600 system (SCIEX, USA) operating in SWATH-MS mode (IonSpray voltage floating 2–200 V, interface heater temperature 150 C). Precursor ions were acquired across an ion mass of 400 – 1–600 m/z with 50 msec accumulation time per spectrum. Variable window widths were used, specifying a maximum of 100 variable windows across a precursor mass range of 400 – 1–200 m/z, with 1 Da window overlap, and minimum window width 4 Da. Rolling collision energy was enabled for each window with 5 eV spread. Fragment ion spectra were accumulated in high sensitivity mode for 30 msec over 100–1800 m/z mass range.

### Proteasome activity assay

To confirm the functionality of encapsulated proteasomes in the EVs, we measured the hydrolysis of 100 μM of the proteasome fluorogenic peptide substrate, Suc-LLVY-AMC (Enzo Life Sciences, USA). EVs samples were sonicated in a bath sonicator for 15 sec (Dekel et al., 2021). In a 96-well black plate, 25 μg of total protein of sonicated EVs was mixed with 100 μM substrate, with or without 10 μM of the proteasome inhibitor MG132 (Enzo Life Sciences, USA), in assay buffer (50 mM Tris, 50 mM NaCl, 5 mM MgCl_2_, 1 mM DTT, pH 7.5) to a total volume of 100 μL. The plate was incubated for 1h in the dark at 37°C. Fluorescence from the hydrolyzed AMC group was measured using a microplate reader (Tecan Infinite 200, excitation 360 nm/emission 460 nm). Final values were obtained by subtracting the blank readings (substrate in assay buffer only).

### Invasion assay with malaria EVs

uRBCs were resuspended to 1% hematocrit with 1× PBS and stained with CellTracker Deep Red (Invitrogen, USA) for 30 min at RT. To stop the staining reaction, fetal bovine serum (FBS) was added followed by 2 washing steps with MCM. A master mix was prepared with stained uRBCs, MCM, and magnet-purified schizont-iRBCs. The master mix was equally distributed in a 96-well flat bottom plate considering duplicates per each condition. EVs from non-treated uRBCs, EVs from non-treated iRBCs and EVs from CQ-treated iRBCs were added containing 300 μg of protein which was calculated using a BCA assay kit (Thermo Fisher Scientific, USA). 1× PBS was used as the control without EVs. For each well, parasites reached 1% parasitemia in 2% hematocrit. The plate was incubated under a gas mixture of 5% CO_2_, 3% O_2_, and 92% N_2_ at 37°C in the dark for 32h. The initial parasitemia was determined by microscopy of Giemsa-stained thin smears using a Nikon Eclipse E800 microscope (Nikon Instruments, USA) and flow cytometry. Right after the addition of the EVs (time 0h), aliquots of each condition were resuspended with 1× PBS and stained with 8 μM Hoechst for 10 min. Two washing steps were performed with 1× PBS. Finally, samples were resuspended with 1× PBS and 100–000 events were immediately acquired on a BD LSR Fortessa X-20 (BD, USA). Giemsa thin smears for microscopy and Hoechst staining for flow cytometry were repeated at 32h.

### Stimulation of THP-1-derived macrophages with malaria EVs

The human monocytic leukemia cell line THP-1 was cultured at 0.5 - 1 × 10^6^ cells/mL in RPMI 1640 Medium, GlutaMAX™ Supplement with 10% FBS (Gibco, Thermo Fisher Scientific, USA) in 25 cm^2^ vented flasks in a humidified incubator at 37°C and 5% CO_2_. THP-1 monocytic cells were transferred to a 12-well flat-bottom plate at 3 x 10^5^ cells/mL. Cells were differentiated using 25 ng/mL phorbol-12-myristate-13-acetate (PMA, Sigma-Aldrich, USA) for 48h to obtain a macrophage-like phenotype. The cells were then washed with fresh culture medium and EVs were added at a final concentration of 300 μg of total protein followed by an incubation of 14h.

### THP-1-derived macrophages cytokine ELISA assays

TNF-α, IL-6 and IL-1β were measured in culture supernatants from THP-1-derived macrophages upon stimulation with EVs or LPS after 14h. Cell culture medium was used as the negative control. These evaluations were performed by the commercial Human ELISA kits (Sigma-Aldrich, USA) according to manufacturer instructions. Experiments were done independently in duplicates. Absorbance was measured by the Infinite^®^ M200 PRO microplate reader (Tecan, Switzerland).

### THP-1-derived macrophages RNA isolation

The medium of the differentiated cells upon stimulation with EVs was carefully removed and kept for cytokine assays. RNA isolation was performed in a sterile area exclusive for RNA work using nuclease free water and RNase-free glass- and plastic ware. Total RNA was isolated by RNAzol (Sigma-Aldrich, USA) following the manufacturer’s protocol. Differentiated cells were mixed with 500 μL of RNAzol to form a homogenous lysate and transferred to 1.5 mL tubes. Immediately, 200 μL of RNase-free water was added into the tubes and mixed well followed by an incubation of 10 min at RT. The samples were centrifuged at 12 000 × g for 15 min at RT to separate the semisolid pellet (containing DNA, proteins, and polysaccharides) from the supernatant (containing RNA). 550 μL of supernatant was transferred into new tubes and mixed with equal volume of isopropanol kept at RT for 10 min. Tubes were centrifuged at 12 000 × g for 10 min at RT to precipitate RNA. RNA pellet was washed twice with 75% ethanol by centrifugation at 6–000 x g for 2 min at RT. The RNA pellet was air-dried and dissolved in 30 μL of nuclease-free water and stored at -80°C until used.

### THP-1-derived macrophages RNA sequencing

The poly(A) mRNA isolation was performed using Oligo(dT) beads. The mRNA fragmentation was performed using divalent cations and high temperature. Priming was performed using Random Primers. First-strand cDNA and the second-strand cDNA were synthesized. The purified double-stranded cDNA was then treated to repair both ends and add a dA-tailing in one reaction, followed by a T-A ligation to add adaptors to both ends. Size selection of Adaptor-ligated DNA was then performed using DNA Clean Beads. Each sample was then amplified by PCR using P5 and P7 primers and the PCR products were validated. Final libraries with different indexes were multiplexed and loaded on a Novaseq PE150 (Illumina, USA) instrument for sequencing using a 2x150 paired-end (PE) configuration according to manufacturer’s instructions.

### Bioinformatic analysis

#### Liquid chromatography - mass spectrometry

Acquired spectra were searched using ProteinPilot v5.0, Paragon algorithm v5.0.0.0, 4–767 against SwissProt Human reference proteome (UP000005640, 2020 Jun release, 20–370 entries) and UniProt *P. falciparum* (isolate 3D7) reference proteome (UP000001450, 2020 Jun release, 5–456 entries), spiked with common contaminant proteins (cRAP). Cysteine alkylation: methyl methane thiosulfonate (MMTS) was applied and biological modifications were enabled. The Venn diagrams were created in https://bioinformatics.psb.ugent.be/webtools/Venn/ to determine the unique and common proteins between samples. KEGG pathways Over-representation analysis were performed using clusterProfiler (v4.4.4) with significant genes classified as adjusted p-value ≤ 0.05 with a minimum of 1.5-fold change.

#### Sequential window acquisition of all theoretical mass spectra

Acquired spectra were searched using the Spectronaut 15.4.210913.50606 (Biognosys AG, Switzerland), DirectDIA workflow against SwissProt Human reference proteome (UP000005640, 2021 Jan release, 20380 entries) and UniProt *P. falciparum* (isolate 3D7) reference proteome (UP000001450, 2020 Aug release, 5–384 entries), spiked with common contaminant proteins (cRAP). The following modifications were used: MMTS at cysteine as a fixed modification, variable oxidation of methionine and acetylation of the protein N-terminus. The enzyme was set as Trypsin, and up to 2 missed cleavages were permitted. The false discovery rate for peptide and protein identifications was set to 1% and at least 95% confidence, the rest of the parameters were set at the default settings. Briefly, the top 10 peptides based on precursor intensity and confidence were selected across the entire retention time (RT) elution range and then scored. The global normalization strategy was based on the median scores. Human and parasite proteome responses were visualized as heatmaps and volcano plots using R package. The latest version of Gene Ontology (GO) annotations for cellular component, biological process and molecular function were obtained as gmt files from gprofiler website (https://biit.cs.ut.ee/gprofiler/gost). The KEGG pathway annotations were mapped directly from KEGG website (https://www.genome.jp/kegg/). Gene Set Enrichment Analysis (GSEA) was performed with R package clusterProfiler (v4.4.4) and the results were visualized with ggplot2. Protein-protein interaction networks were created by submitting the UniProt ascensions to the STRING (Search Tool for the Retrieval of Interacting Genes) software (http://string-db.org/). Interaction networks were visualized for proteins with medium confidence (0.4) with network edges based on evidence, with continuous lines for direct interactions. Clustering was based on an MCL algorithm inflation default parameter of 3.

#### RNA sequencing - THP-1-derived macrophages

To remove technical sequences and quality of bases lower than 20, pass filter data were processed by Cutadapt (v1.9.1) to be high-quality clean data. Afterward, the data was aligned to the reference genome via the software Hisat2 (v2.0.1). For the expression analysis, HTSeq (v0.6.1) was used to estimate gene and isoform expression levels from the pair-end clean data. The differential expression analysis was performed by DESeq2 (v1.36.0) Bioconductor package, adjusted p-value of genes were set <0.05 to detect differentially expressed ones. Gene Set Enrichment Analysis (GSEA) was performed with R package clusterProfiler (v4.4.4). Volcano plots with pathway annotations were made with ggplot2 (v3.3.6) and shiny(v1.7.2).

### Statistical analysis

Statistical analysis was performed using FlowJo™ v10 (FlowJo Software, USA) and GraphPad Prism v9 (GraphPad Software, USA). Kruskal-Wallis test and two-way analysis of variance (ANOVA) were used to evaluate experiments involving multiple groups. The test used is indicated in the figure legends. Graphs show mean ± sem * p < 0.05, ** p < 0.01, *** p < 0.001, **** p < 0.0001.

## Data Availability

The database for mass spectrometry and RNA sequencing can be found here: https://data.mendeley.com/datasets/v4fgbc6t8r/1.
