# Supplementary material for: Chloroquine induces eryptosis in P. falciparum-infected red blood cells and the release of extracellular vesicles with a unique protein profile
Source: Front Cell Infect Microbiol. 2025 May 26;15:1553123. doi: 10.3389/fcimb.2025.1553123 (PMC12146344; doi:10.3389/fcimb.2025.1553123)
Supplement: Supplementary file 1 [file DataSheet1.docx]

**SUPPLEMENTAL FIGURES LEGENDS**

**Supplemental Fig. 1. NTA size distribution analysis.** A representative NTA plot per each sample is shown with the mean number size distribution of EVs. The analysis is based on Brownian motion, detected through the light scattering signal. All liquid dispersions were diluted 1000-fold and measured in quintuplicates.

**Supplemental Fig. 2. GO analysis of human and parasite proteins from EVs.** Cellular component, biological process and molecular function for (A-C) human and (D-F) parasite proteins. The results represent 3 biological replicates per sample. Only pathways that passed the threshold (fold change ≥ 1.5, adjusted p-value ≤ 0.05) are shown in the bubble plots. ND: EVs from non-treated iRBCs; CQ: EVs from CQ-treated iRBCs; MQ: EVs from MQ-treated iRBCs.

**Supplemental Fig. 3. GO and KEGG analysis of CQ-treated iRBCs lysates.** Human proteins from CQ-treated iRBCs (CQ) in contrast with non-treated iRBCs (Control) were analysed for (A) GO-Cellular component and (B) KEGG enrichment pathways. Parasite proteins from CQ-treated iRBCs in contrast with non-treated iRBCs were analysed for (C) GO-Cellular component and (D) KEGG enrichment pathways. The results represent 3 biological replicates per sample. Only pathways that passed the threshold (fold change ≥ 1.5, adjusted p-value ≤ 0.05) are shown in the bubble plots.

**Supplemental Fig. 4. Proteasome proteolytic activity measurement of iRBCs-derived EVs with the fluorescent peptide Suc-LLVY-AMC**. uRBC-derived EVs and the proteasome inhibitor MG132 were used as controls. The data was subtracted to the background level of fluorescence in the assay buffer. Statistical analysis was performed on 4 biological replicates using two-way ANOVA, Tukey’s multiple comparison test.

**Supplemental Fig. 5. iRBCs-derived EVs effect in parasite growth rate.** (A) Giemsa smears of invasion assays at T0h and T32h. 1X PBS used to resuspend EV samples was included as the control. Scale bars = 10 μm. (B) Parasite growth rate normalized to the control from microscopy and flow cytometry data. The results are represented in mean ±  s.e.m. of 3 independent experiments by Kruskal-Wallis test with post-hoc Dunn’s test. No significant difference was found.

**Supplemental Fig. 6. ELISA assay for detection of TNF-α, IL-6 and IL-1β in THP-1 derived-macrophages.** All samples were compared to the control (media without EVs). LPS was used as a positive control. The results are represented in mean ± SEM of 3 independent experiments; **p* < 0.05, *****p* < 0.0001 by Kruskal-Wallis test with post-hoc Dunn’s test.

**Supplemental Fig. 7. GO analysis of THP-1-derived macrophages upon stimulation with EVs.** (A) Cellular component, (B) biological process and (C) molecular function. The results represent 3 biological replicates per sample. Only pathways that passed the threshold (fold change ≥ 1.5, adjusted p-value ≤ 0.05) are shown in the bubble plots.
