# Supplementary figures and images for: Chloroquine induces eryptosis in P. falciparum-infected red blood cells and the release of extracellular vesicles with a unique protein profile
Source: Front Cell Infect Microbiol. 2025 May 26;15:1553123. doi: 10.3389/fcimb.2025.1553123 (PMC12146344; doi:10.3389/fcimb.2025.1553123)

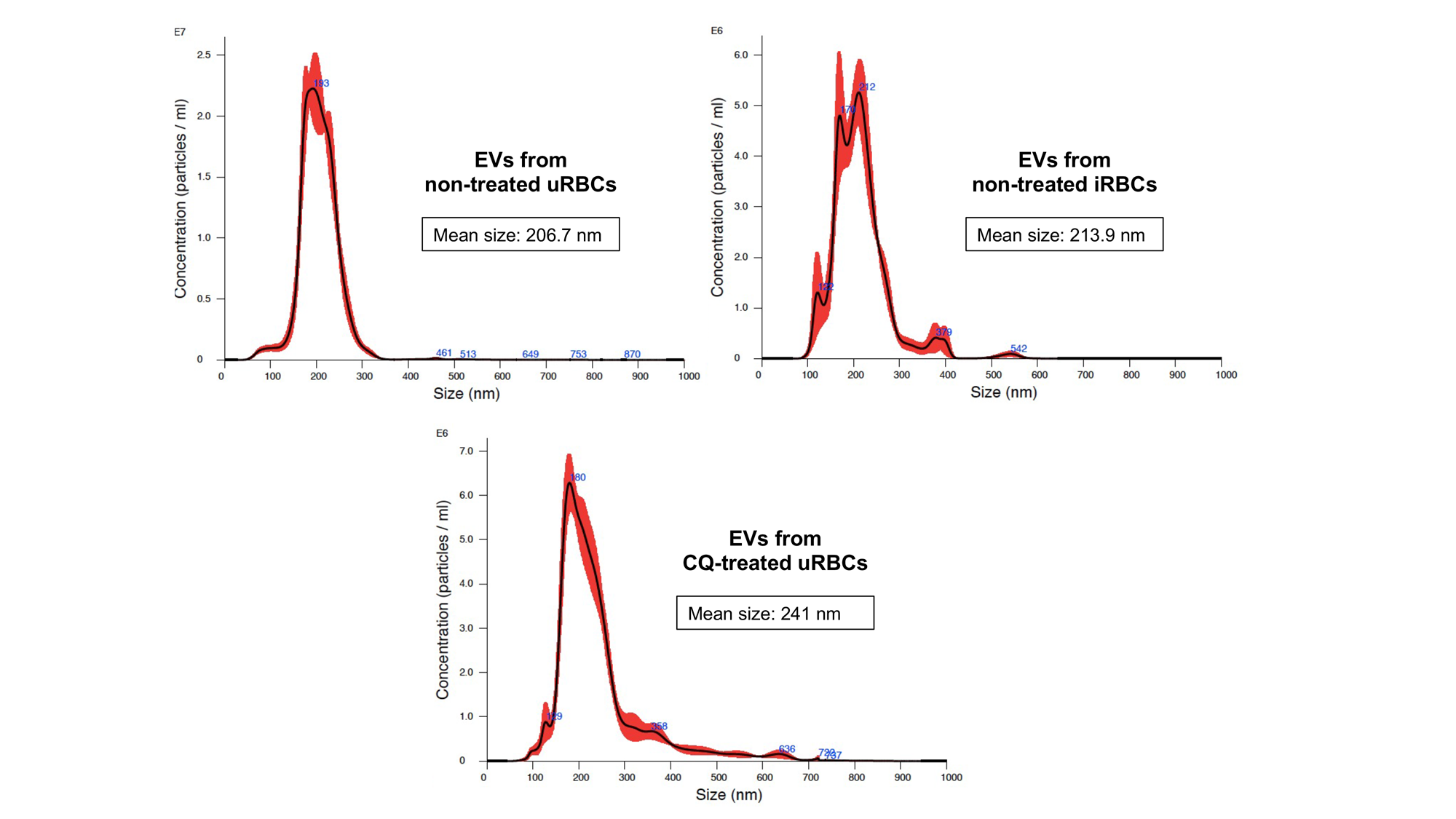

Supplement: Supplementary file 3 [file Image1.tif]

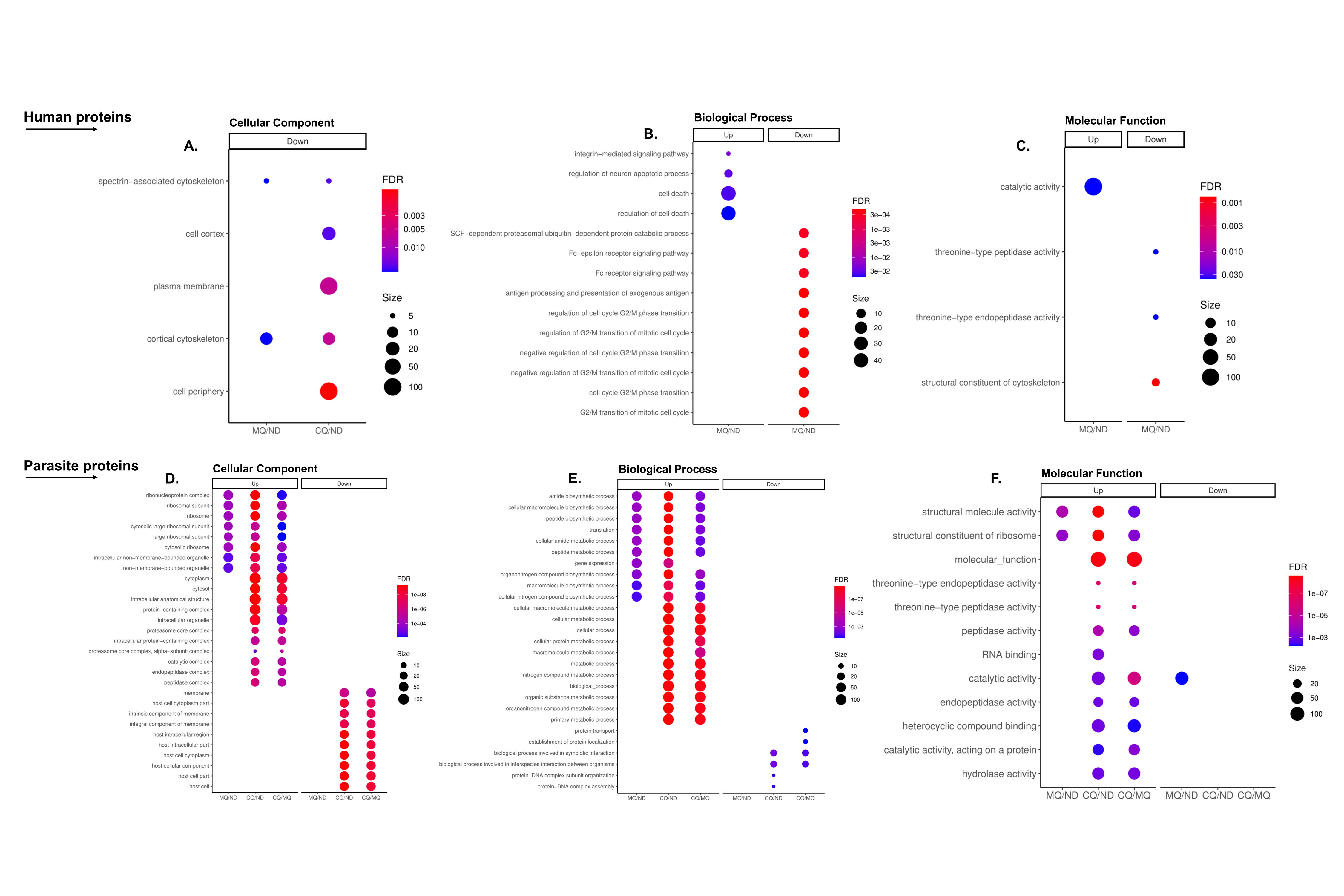

Supplement: Supplementary file 4 [file Image2.tif]

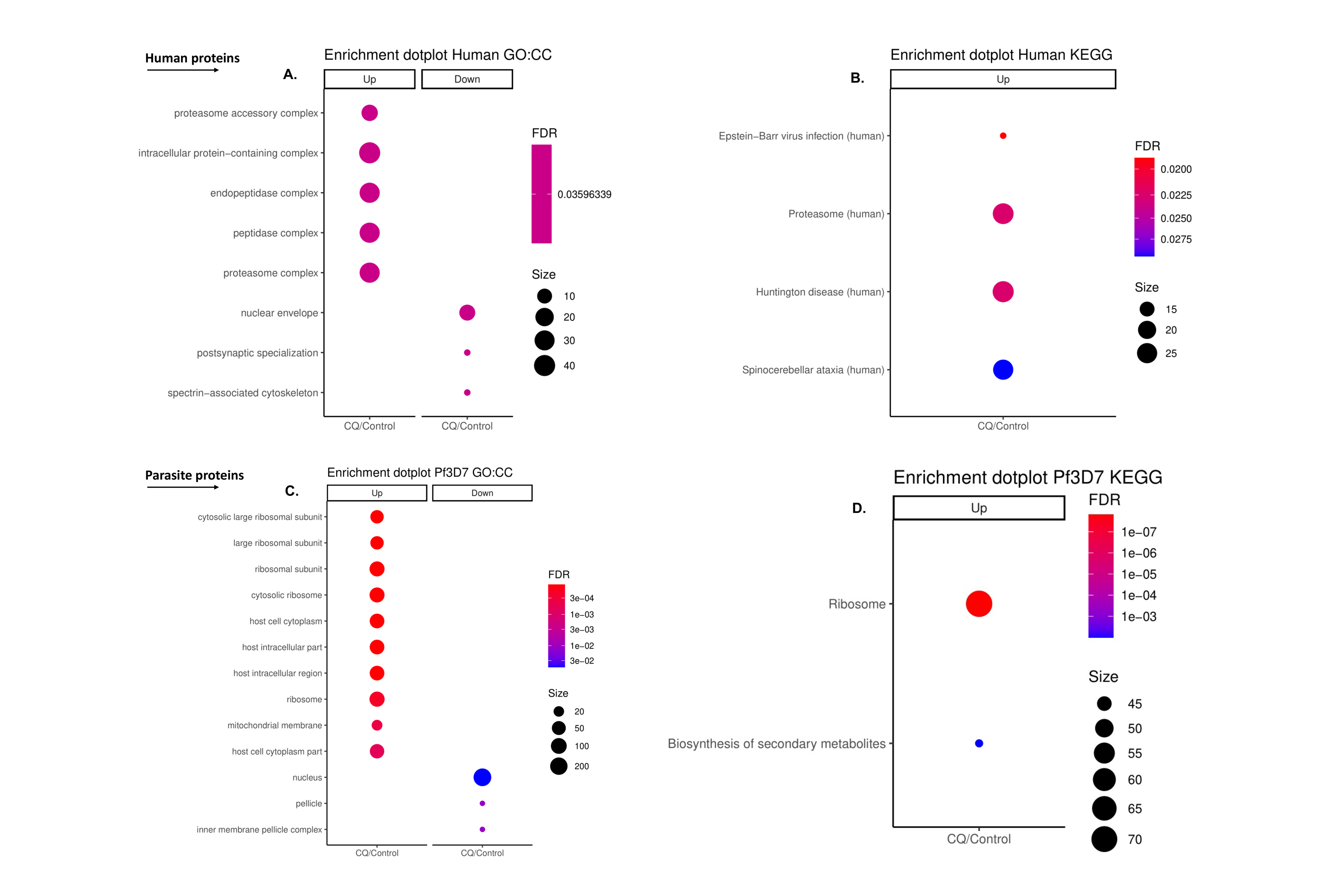

Supplement: Supplementary file 5 [file Image3.tif]

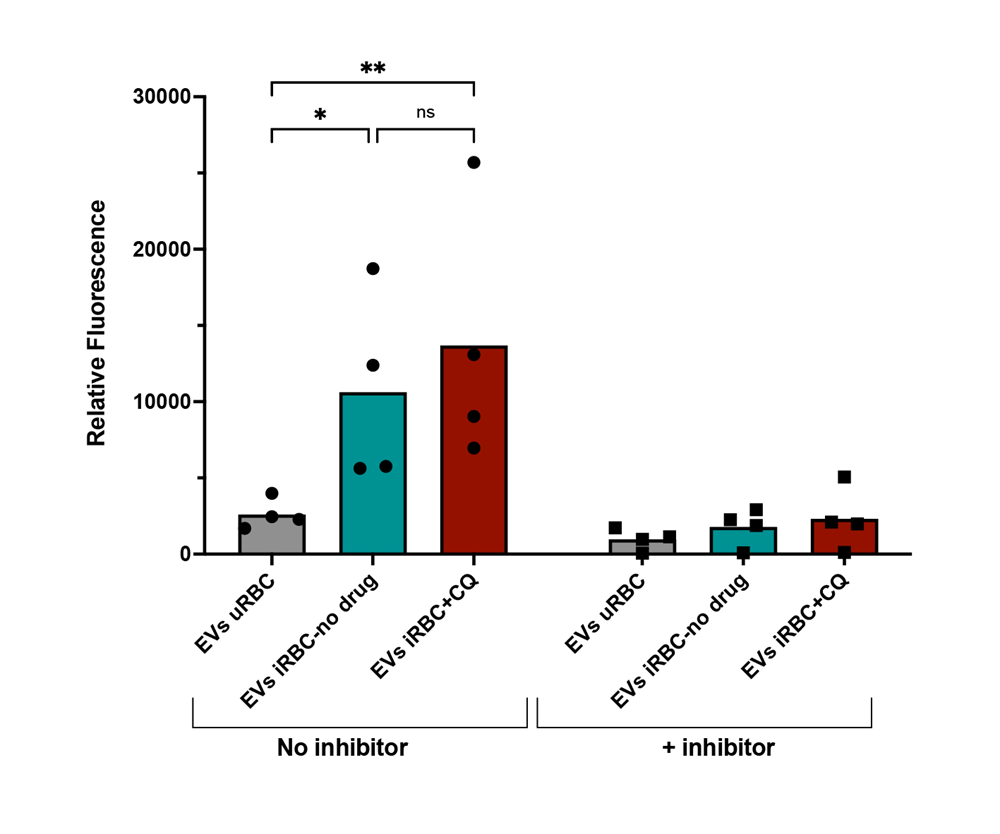

Supplement: Supplementary file 6 [file Image4.tif]

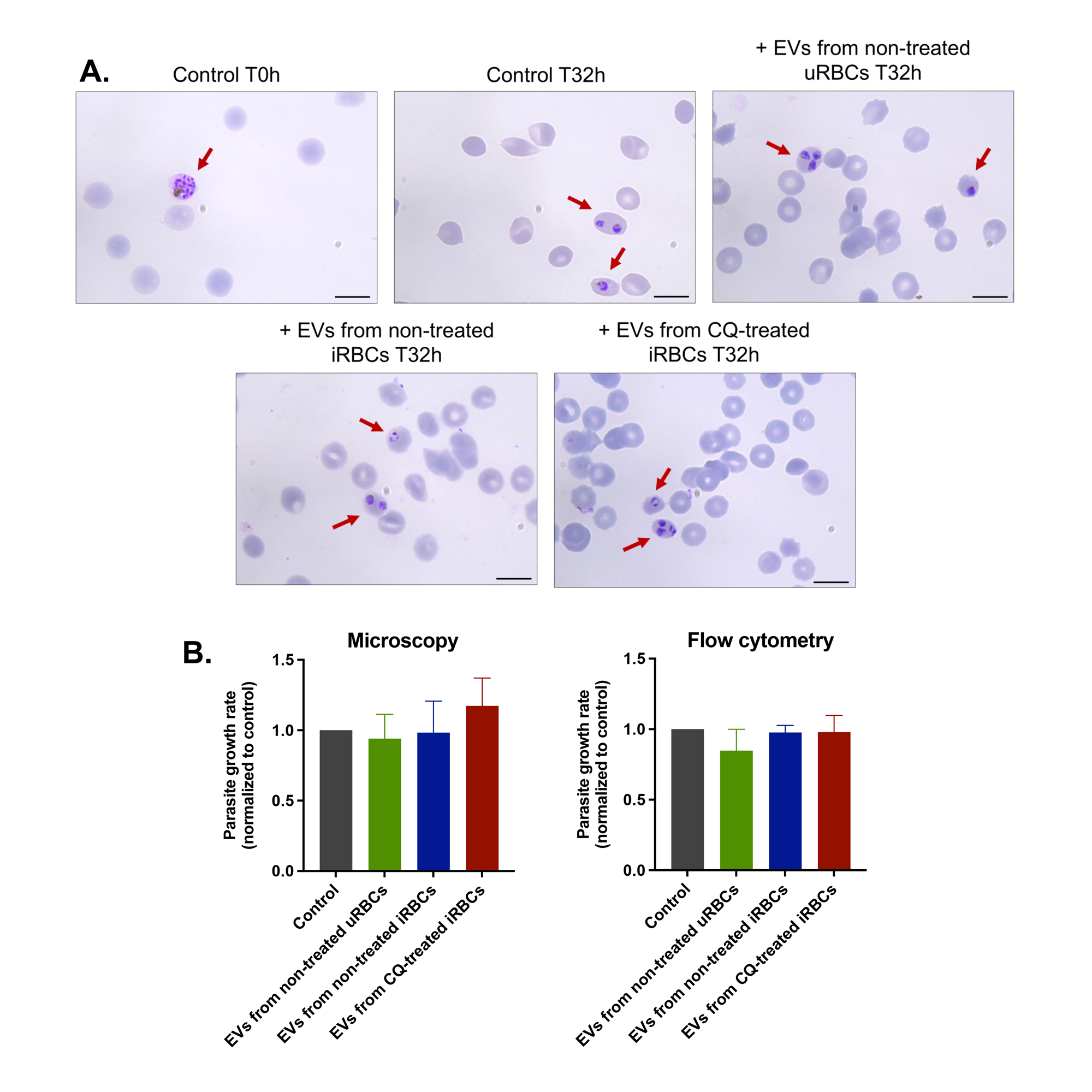

Supplement: Supplementary file 7 [file Image5.tif]

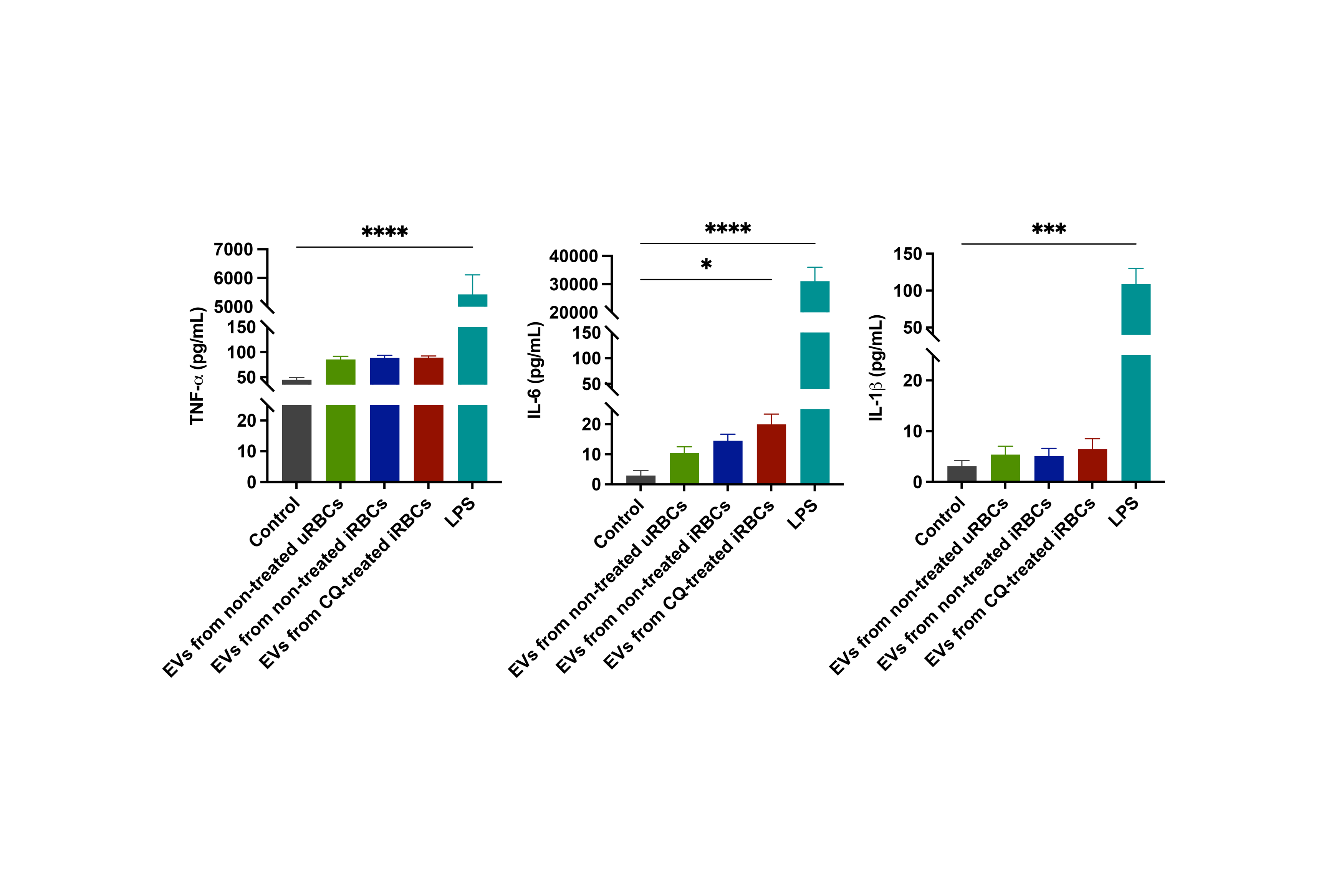

Supplement: Supplementary file 8 [file Image6.tif]

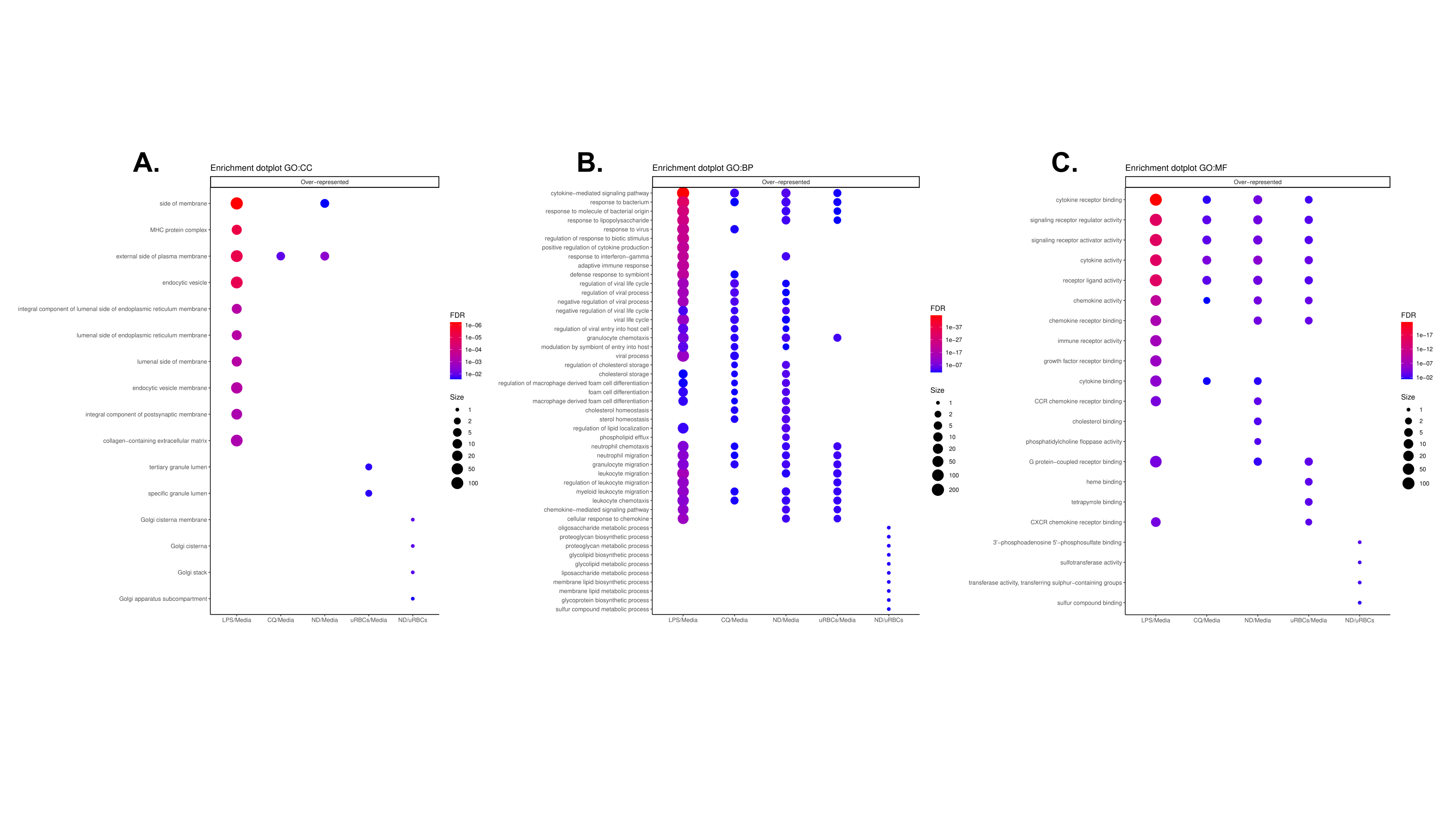

Supplement: Supplementary file 9 [file Image7.tif]
